# Novel Partitivirus Enhances Virulence of and Causes Aberrant Gene Expression in Talaromyces marneffei

**DOI:** 10.1128/mBio.00947-18

**Published:** 2018-06-12

**Authors:** Susanna K. P. Lau, George C. S. Lo, Franklin W. N. Chow, Rachel Y. Y. Fan, James J. Cai, Kwok-Yung Yuen, Patrick C. Y. Woo

**Affiliations:** a State Key Laboratory of Emerging Infectious Diseases, The University of Hong Kong, Hong Kong, China; b Research Centre of Infection and Immunology, The University of Hong Kong, Hong Kong, China; c Carol Yu Centre for Infection, The University of Hong Kong, Hong Kong, China; d Department of Microbiology, LKS Faculty of Medicine, The University of Hong Kong, Hong Kong, China; e Collaborative Innovation Center for Diagnosis and Treatment of Infectious Diseases, The University of Hong Kong, Hong Kong, China; f Department of Veterinary Integrative Biosciences, Texas A&M University, College Station, Texas, USA; University of California, Berkeley

**Keywords:** *Talaromyces marneffei*, dimorphic, fungus, novel, partitivirus

## Abstract

Talaromyces marneffei is the most important thermal dimorphic fungus causing systemic mycosis in Southeast Asia. We report the discovery of a novel partitivirus, Talaromyces marneffei *partitivirus*-1 (TmPV1). TmPV1 was detected in 7 (12.7%) of 55 clinical T. marneffei isolates. Complete genome sequencing of the seven TmPV1 isolates revealed two double-stranded RNA (dsRNA) segments encoding RNA-dependent RNA polymerase (RdRp) and capsid protein, respectively. Phylogenetic analysis showed that TmPV1 occupied a distinct clade among the members of the genus *Gammapartitivirus*. Transmission electron microscopy confirmed the presence of isometric, nonenveloped viral particles of 30 to 45 nm in diameter, compatible with partitiviruses, in TmPV1-infected T. marneffei. Quantitative reverse transcription-PCR (qRT-PCR) demonstrated higher viral load of TmPV1 in the yeast phase than in the mycelial phase of T. marneffei. Two virus-free isolates, PM1 and PM41, were successfully infected by purified TmPV1 using protoplast transfection. Mice challenged with TmPV1-infected T. marneffei isolates showed significantly shortened survival time (*P* < 0.0001) and higher fungal burden in organs than mice challenged with isogenic TmPV1-free isolates. Transcriptomic analysis showed that TmPV1 causes aberrant expression of various genes in T. marneffei, with upregulation of potential virulence factors and suppression of RNA interference (RNAi)-related genes. This is the first report of a mycovirus in a thermally dimorphic fungus. Further studies are required to ascertain the mechanism whereby TmPV1 enhances the virulence of T. marneffei in mice and the potential role of RNAi-related genes in antiviral defense in T. marneffei.

## IMPORTANCE

Talaromyces marneffei (formerly Penicillium marneffei) is the most important thermal dimorphic fungus in Southeast Asia, causing highly fatal systemic penicilliosis in HIV-infected and immunocompromised patients. We discovered a novel mycovirus, TmPV1, in seven clinical isolates of T. marneffei. TmPV1 belongs to the genus *Gammapartitivirus* of the family *Partitiviridae*. We showed that TmPV1 enhanced the virulence of T. marneffei in mice, with shortened survival time and higher fungal burden in the organs of mice challenged with TmPV1-infected T. marneffei isolates than in those of mice challenged with virus-free isogenic isolates. Transcriptomics analysis showed that TmPV1 altered the expression of genes involved in various cellular processes in T. marneffei, with upregulation of potential virulence factors and suppression of RNAi machinery which may be involved in antiviral defense. This is the first report of a mycovirus in a thermal dimorphic fungus. The present results offer insights into mycovirus-fungus interactions and pathogenesis of thermal dimorphic fungi.

## INTRODUCTION

Mycoviruses are present in a wide variety of fungi. Those with double-stranded RNA (dsRNA) genomes are now classified into 12 families, including *Amalgaviridae*, *Birnaviridae*, *Chrysoviridae*, *Cystoviridae*, *Endornaviridae*, *Hypoviridae*, *Megabirnaviridae*, *Partitiviridae*, *Picobirnaviridae*, *Quadriviridae*, *Reoviridae*, and *Totiviridae* (http://ictvonline.org/). Most mycoviruses cause cryptic infections, but some mycoviruses, including dsRNA and single-stranded RNA and DNA viruses, are associated with phenotypic alterations such as hypovirulence and debilitation in their hosts (1–5). As a result, mycoviruses have been utilized for disease control in plants (6–8). On the other hand, some dsRNA viruses were found to confer a survival advantage to their hosts. For example, totiviruses can enhance the survival of Saccharomyces cerevisiae by encoding a killer toxin (Klus) with broad antifungal activity that can inhibit competitive fungi such as virus-free S. cerevisiae isolates (9, 10). Despite their ubiquitous nature, studies on mycoviruses in human-pathogenic fungi are scarce. In *Aspergillus*, dsRNA mycoviruses are common in several asexual species, though with wide variations in number and composition within a species, resulting in infection rates ranging from 0% to 13% (11–14). Mycovirus infection of Aspergillus fumigatus may result in aberrant phenotype alternations and growth attenuation (3). Recently, a potentially novel A76 mycovirus that infects A. fumigatus was found to enhance the fungal virulence in a moth larvae model (15). However, little is known about the biological role of mycoviruses in other human-pathogenic fungi and the potential mechanisms by which mycoviruses may alter fungal virulence.

Talaromyces marneffei, previously known as Penicillium marneffei, is the most important thermal dimorphic fungus causing respiratory, skin, and systemic mycosis in Southeast Asia (16–19). The fungus was first discovered in 1956 in isolates from Chinese bamboo rats, Rhizomys sinensis (20, 21). T. marneffei infection disease, also known as penicilliosis, was once considered a rare disease, with only 18 cases of human diseases reported by 1985 (22). However, increasing reports of HIV-associated penicilliosis were noted in Southeast Asia since the emergence of the HIV pandemic in the 1980s. In countries such as northern Thailand, penicilliosis is among the top three most common indicator diseases of acquired immunodeficiency syndromes, together with tuberculosis and cryptococcosis (17). In Hong Kong, up to 8% of HIV-infected patients have been infected with T. marneffei, which represents the sixth leading cause of deaths (23, 24). Moreover, T. marneffei infections are increasingly reported in other immunocompromised hosts such as transplant recipients and other patients receiving corticosteroid therapy and immunotherapy (25–28). Imported cases of penicilliosis have also been reported in countries where the disease is not endemic (29, 30).

Despite recent advances in understanding the epidemiology and virulence factors of T. marneffei through molecular and genomic studies (31–37), various aspects of its biology, transmission, and pathogenesis remain largely unknown. Moreover, no mycoviruses have been reported in T. marneffei or in other thermally dimorphic fungi that are important human pathogens. Since mycoviruses have been shown to alter the virulence of fungi, we hypothesize that mycoviruses may infect T. marneffei and may play a role in the fungal virulence. In this report, we describe the discovery and genomic characterization of the first mycovirus, Talaromyces marneffei *partitivirus*-1 (TmPV1), in T. marneffei, which can be detected in both the mycelial and yeast phases. Using isogenic virus-infected and virus-free T. marneffei isolates, we showed that TmPV1 enhanced the virulence of T. marneffei in infected mice and may suppress the expression of RNA interference (RNAi)-related genes. Transcriptomics studies revealed that TmPV1 may upregulate genes that are potential virulence factors in T. marneffei.

## RESULTS

### dsRNA segments in T. marneffei.

**FIG 1:**
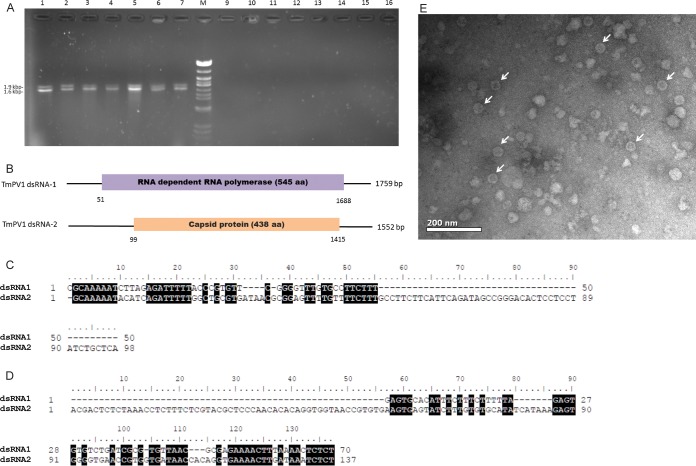
(A) dsRNA extracted from Talaromyces marneffei. (B) Schematic representation of the genome organization of TmPV1 dsRNA-1 and dsRNA-2. (C and D) Conserved 5′-UTR (C) and 3′-UTR (D) sequence elements in TmPV1 dsRNA-1 (top rows) and dsRNA-2 (bottom rows). (E) Transmission electron microscopy of TmPV1 purified from T. marneffei isolate PM40. (A) Lanes 1 to 7, two dsRNA bands were observed in 7 T. marneffei isolates; lanes 9 to 16, no dsRNA bands were observed in the remaining 48 T. marneffei isolates (7 selected isolates are shown); lane M, lambda DNA digested by Eco471 (AvaII). Sizes are indicated as kilobase pairs (left). (B) Open bars represent open reading frames (ORFs) encoding the putative RNA-dependent RNA polymerase or capsid protein. The 5′ and 3′ untranslated regions are indicated as single lines. The nucleotide positions of the initiation and termination codons are indicated below the border of the ORF. Nucleotides in the 5′-UTR (C) and 3′-UTR (D) of dsRNA-1 and dsRNA-2 that are identical are indicated with reverse highlighting. (E) Isometric, nonenveloped viral particles of 30 to 45 nm in diameter, compatible with partitiviruses, are indicated by white arrows. Bar = 200 nm.

dsRNAs isolated from the mycelial mass of 55 T. marneffei isolates were subjected to agarose gel electrophoresis. Two distinct bands consistent with partitiviruses, one at approximately 1.9 kb and the other at 1.6 kb, were observed from seven T. marneffei isolates under UV light (Fig. 1). However, no bands were observed in the other 48 isolates. Further testing using dsRNA isolated from yeast cultures of the seven positive isolates also showed the presence of the two distinct bands, suggesting that the potential partitivirus was present in both the mycelial and yeast phases of the infected T. marneffei isolates.

### Nucleotide sequence and genome organization of TmPV1.

The dsRNA segments from the seven T. marneffei isolates were subjected to gel purification and to cDNA cloning with random reverse transcription-PCR (RT-PCR) amplification. Cloning and sequencing showed that the two dsRNA bands represented two distinct dsRNA segments, dsRNA-1 and dsRNA-2, characteristic of partitiviruses. The 1.9-kb fragment showed sequence similarity to the RNA-dependent RNA polymerase (RdRp) genes of partitiviruses (family *Partitiviridae*), whereas the 1.6-kb fragment showed sequence similarity to the capsid genes of partitiviruses. This suggests that the two dsRNA segments represent a putative partitivirus, which is proposed to be named Talaromyces marneffei *partitivirus*-1 (TmPV1).

The complete genome sequences of TmPV1 from the seven T. marneffei isolates were obtained by a combination of PCR and cloning techniques. Partitiviruses typically have bipartite genomes consisting of two major monocistronic dsRNA segments of 1.0 to 3.0 kbp, each containing a single open reading frame (ORF). The genome organization of TmPV1 is typical of partitiviruses (38), with two dsRNA fragments that are referred to dsRNA-1 (1,759 bases) and dsRNA-2 (1,552 bases), respectively (Fig. 1). The overall base compositions of TmPV1 dsRNA-1 were 24.3% A, 27.0% G, 26.5% T(U), and 22.2% C, and the overall base compositions of TmPV1 dsRNA-2 were 20.6% A, 27.2% G, 25.9% T(U), and 26.3% C. Each dsRNA contains 5′ untranslated regions (5′-UTRs) and 3′-UTRs and a single ORF on its plus-strand RNA. No ORFs were predicted in the minus-strand RNAs. The 5′-UTRs and 3′-UTRs of TmPV1 dsRNA-1 were 50 and 70 bases long and those of TmPV1 dsRNA-2 were 98 and 137 bases long. The two 5′-UTRs shared sequence identity of 33%, with identical 5′-CGAAAAAU-3′ terminal motifs and a stretch of conserved sequences with 33 identical nucleotides (Fig. 1). The 5′-UTR of dsRNA-1 was most closely related to that of Penicillium stoloniferum *virus* S (PsV-S) (GenBank accession number AM040148.1), with 62.7% nucleotide identity, while the 5′-UTR of dsRNA-2 was most closely related to that of Ustilaginoidea virens partitivirus (GenBank accession number KC503899.1), with 62.6% nucleotide identity. The 5′-UTRs of both TmPV1 dsRNA-1 and dsRNA-2 were A/U rich, but they were devoid of CAA repeats, which are characteristic of some members of the partitiviruses (39). The 3′-UTRs of both TmPV1 dsRNA-1 and dsRNA-2 also contained A/U-rich sequences, but, unlike some partitiviruses (40–42), they did not contain a poly(A) tail. They shared stretches of conserved sequences, including the 5′-UCUCU-3′ terminal motif (Fig. 1). The seven isolates of TmPV1 shared highly similar genome sequences, with 96.5% to 99.7% nucleotide identities.

### Sequence and phylogenetic analyses of predicted proteins of TmPV1.

**FIG 2:**
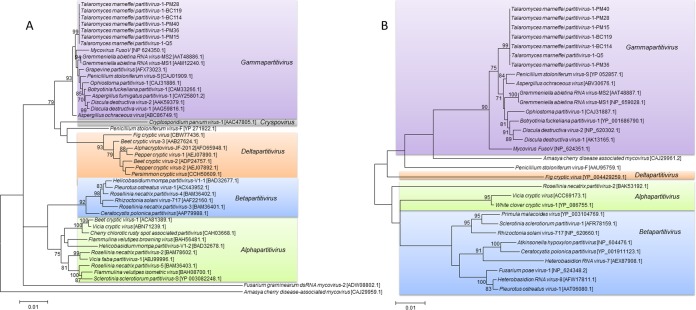
Phylogenetic tree constructed from the amino acid sequences of the RdRp (A) and capsid proteins (B) of TmPV1 and other members of *Partitiviridae*. Totals of 360 and 801 amino acid positions were included in the analysis, respectively. Phylogenetic analysis was performed by maximum likelihood based on the Le_Gascuel_2008 model with gamma distributions (A) and the General Reverse Transcriptase with Frequency model, gamma distributions (B), using MEGA 6. Bootstrap values were calculated as percentages from 100 replicates, and only values of ≥70% are shown. The scale bars indicate the estimated number of substitutions per 100 amino acids. Accession numbers are given as cited in the GenBank database.

TmPV1 dsRNA-1 contained a single ORF (nucleotide positions 51 to 1688) potentially encoding a protein of 545 amino acid residues with a molecular mass of 62.8 kDa (Fig. 1). The predicted protein in dsRNA-1 shared sequence similarity with RdRp proteins of other partitiviruses, with 74% amino acid identity to that of *Grapevine partitivirus* (GPV) (GenBank accession number AFX73023.1), 73.3% amino acid identity to that of PsV-S (GenBank accession number YP_052856.2), and 71.8% amino acid identity to that of Aspergillus ochraceus *virus* (AoV) (GenBank accession number ABC86749.1) (see Table S1 in the supplemental material). While the N terminus of the predicted RdRp of TmPV1 showed low sequence homologies to that of other partitiviruses, the central and C-terminal regions shared significant homologies to the corresponding regions in other partitiviruses and possessed conserved motifs characteristic of RdRp sequences of other members of the *Partitiviridae* (see Fig. S1 in the supplemental material) (41, 43, 44). These conserved motifs include the reverse transcriptase-like superfamily domain, active site, nucleoside triphosphate (NTP) binding site, and nucleic acid binding site. Phylogenetic analysis showed that the RdRp of TmPV1 was most closely related to the RdRp of members of the genus *Gammapartitivirus*, with sequences from the seven T. marneffei isolates forming a distinct clade within the genus with a high bootstrap value of 100 (Fig. 2). The seven TmPV1 isolates shared 96.4% to 99.6% amino acid identities in the RdRp.

10.1128/mBio.00947-18.1 FIG S1  Amino acid sequence alignment of the (A) RdRp and (B) capsid proteins of TmPV1 and related partitiviruses PsV-S, AoV, GaV-MS2, GaV-MS1, BfPV-1, AfPV, DdV-1, GPV, DdV-2, OPV-1, FusoV, and PsV-F. Conserved amino acids are indicated with reverse highlighting. For RdRp, regions marked with the solid line on the top indicate the reverse transcriptase-like superfamily domain. The region underlined with the dotted line indicates the active site. Sites marked with a crest (^) indicate the NTP binding site. The asterisk (*) indicates the nucleic acid binding site. TmPV1, Talaromyces marneffei partitivirus-1; PsV-S, Penicillium stoloniferum virus-S; AoV, Aspergillus ochraceus virus; GaV-MS2, Gremmeniella abietina RNA virus-MS2; GaV-MS1, Gremmeniella abietina RNA virus-MS1; BfPV-1, Botryotinia fuckeliana partitivirus-1; AfPV, Aspergillus fumigatus partitivirus-1; DdV-1, Discula destructiva virus-1; GPV, *Grapevine partitivirus*; DdV-2, Discula destructiva virus-2; OPV-1, Ophiostoma partitivirus-1; FusoV, mycovirus FusoV; PsV-F, Penicillium stoloniferum virus-F. Download FIG S1, TIF file, 4.1 MB. Copyright © 2018 Lau et al. 2018 Lau et al. This content is distributed under the terms of the Creative Commons Attribution 4.0 International license.

10.1128/mBio.00947-18.7 TABLE S1  Homology of the predicted amino acid sequence of TmPV1 RdRp and capsid protein with corresponding regions of other *Partitiviridae*. Download TABLE S1, DOCX file, 0.02 MB. Copyright © 2018 Lau et al. 2018 Lau et al. This content is distributed under the terms of the Creative Commons Attribution 4.0 International license.

TmPV1 dsRNA-2 contained a single ORF (nucleotide positions 99 to 1415) potentially encoding a protein of 438 amino acid residues with a molecular mass of 47.3 kDa (Fig. 1). The predicted protein in dsRNA-2 shared sequence similarity with capsid proteins of other partitiviruses, with 55.9% amino acid identity to that of PsV-S (GenBank accession number YP_052857.1), 54.9% amino acid identity to that of AoV (GenBank accession number ABV30676.1), and 51.4% amino acid identity to that of Botryotinia fuckeliana *partitivirus-*1 (BfPV-1) (GenBank accession number YP_001686790.1) (Table S1). As no capsid protein sequence is available for GPV, whether the capsid of TmPV1 may also be closely related to that of GPV remains undetermined. Phylogenetic analysis showed that the capsid protein of TmPV1 was most closely related to the capsid proteins of members of the genus *Gammapartitivirus*, with sequences from the seven T. marneffei isolates also forming a distinct clade within the genus with high bootstrap value of 100 (Fig. 2). The seven TmPV1 isolates shared 96.6% to 99.5% amino acid identities in the capsid proteins. The present results supported the classification of TmPV1 as a new species of the genus *Gammapartitivirus* under the family *Partitiviridae*.

### Purification and characterization of viral particles.

Viral particles were purified from a TmPV1-infected T. marneffei isolate, PM40, and subjected to transmission electron microscopy. Isometric, nonenveloped, spherical viral particles of 30 to 45 nm in diameter were observed, compatible with other members of *Partitiviridae* (Fig. 1) (39). Purified viral particle fractions were then used for dsRNA extraction. Distinct dsRNAs that had the same sizes as those described above for TmPV1 were observed. The purified viral genome was resistant to DNase and to RNase A at a high salt concentration (2× saline–sodium citrate [SSC] buffer [1× SSC is 0.3 M NaCl plus 0.03 M sodium citrate]). However, it was completely degraded by treatment with RNase A at a low salt concentration (0.1× SSC). Moreover, reverse transcription-PCR (RT-PCR) results obtained using TmPV1-specific primers were positive for bands of the expected size.

### Detection of TmPV1 in the mycelial phase and yeast phase of T. marneffei isolates by quantitative reverse transcription-PCR (qRT-PCR).

In all seven TmPV1-infected T. marneffei isolates, qRT-PCR results for the viral capsid protein gene were positive in both the mycelial and yeast phases. The viral load of TmPV1 in the yeast phase was significantly higher than that in the mycelial phase by approximately 2-fold to 8-fold (*P* < 0.05 by Student’s *t* test) (Fig. S2).

10.1128/mBio.00947-18.2 FIG S2  Relative viral quantities of TmPV1 in the mycelial phase and yeast phase of the seven naturally infected T. marneffei isolates. Results were obtained from four independent experimental replicates. Statistical analysis was performed between the mycelial phase and yeast phase of each T. marneffei isolate using Student’s *t* test. ***, *P* < 0.0001. Error bars indicate standard errors of the means. Download FIG S2, TIF file, 0.3 MB. Copyright © 2018 Lau et al. 2018 Lau et al. This content is distributed under the terms of the Creative Commons Attribution 4.0 International license.

### Effects of TmPV1 infection on fungal virulence.

To elucidate the potential biological function of TmPV1 in T. marneffei, two virus-free isolates, PM1 and PM41, were subjected to TmPV1 infection using purified viral particles and protoplast transfection to obtain isogenic virus-infected PM1 and PM41 isolates, respectively. The virus-infected isolates were confirmed to be positive for TmPV1 RdRp and capsid genes by RT-PCR assay. Amplification and sequencing of the isolate-specific *mp1* gene confirmed that they were isogenic isolates derived from PM1 and PM41, respectively. No significant differences in colony morphologies and microscopic features in both the yeast and mycelial phases or in mycelial growth rates were observed between virus-free and virus-infected isogenic T. marneffei isolates (Fig. S3). No significant differences in scanning electron microscopy features were observed between virus-free and virus-infected isogenic T. marneffei isolates (Fig. S4). Comparisons of intracellular survival rates of T. marneffei in murine macrophage J774 also showed no differences between virus-free and virus-infected isogenic isolates (Fig. S5).

10.1128/mBio.00947-18.3 FIG S3  (A to P) Effects of TmPV1 infection on fungal morphology. (Q to R) Comparison of growth rates of TmPV1-infected and TmPV1-free isogenic T. marneffei isolates of PM1 and PM41. (A to H) Macroscopic appearance of fungal colonies of TmPV1-free and isogenic TmPV1-infected PM1 and PM41 in the mycelial phase and yeast phase. (I to P) Microscopic appearance of TmPV1-free and isogenic TmPV1-infected PM1 and PM41 in mycelial and yeast (magnification, ×400). Fungal elements were stained using lactophenol cotton blue. No morphological changes were observed in comparisons between TmPV1-free and TmPV1-infected isogenic T. marneffei isolates at both the macroscopic and microscopic levels. (Q to R) A total of 500 viable spores of each isolate were inoculated on SDA, and the diameters of the colonies were measured and recorded. No significant differences between TmPV1-infected and TmPV1-free isogenic isolates were observed. Error bars indicate means ± standard errors (SE). Download FIG S3, TIF file, 11.8 MB. Copyright © 2018 Lau et al. 2018 Lau et al. This content is distributed under the terms of the Creative Commons Attribution 4.0 International license.

10.1128/mBio.00947-18.4 FIG S4  Scanning electron microscopy of TmPV1-infected and virus-free T. marneffei. Fungal samples were mounted on aluminum stubs and coated with palladium. Coated samples were observed under a Hitachi S-3400N variable-pressure scanning electron microscope. No significant changes were observed in the cell wall in comparisons between TmPV1-free and TmPV1-infected isogenic T. marneffei isolates at both the mycelial and yeast phases. Download FIG S4, TIF file, 14.3 MB. Copyright © 2018 Lau et al. 2018 Lau et al. This content is distributed under the terms of the Creative Commons Attribution 4.0 International license.

10.1128/mBio.00947-18.5 FIG S5  Intracellular survival of TmPV1-infected and TmPV1-free isogenic PM1 isolates (A) and PM41 isolates (B) in J774 murine macrophages. The cells were inoculated with the conidia at a MOI of 1 and incubated for 2 h to obtain the baseline number of the engulfed viable conidia (0 h). The cells were further incubated for 24 h after removal of the extracellular conidia, and the number of CFU recovered was obtained. No significant differences between TmPV1-infected and TmPV1-free isogenic isolates were observed. Download FIG S5, TIF file, 0.9 MB. Copyright © 2018 Lau et al. 2018 Lau et al. This content is distributed under the terms of the Creative Commons Attribution 4.0 International license.

**FIG 3:**
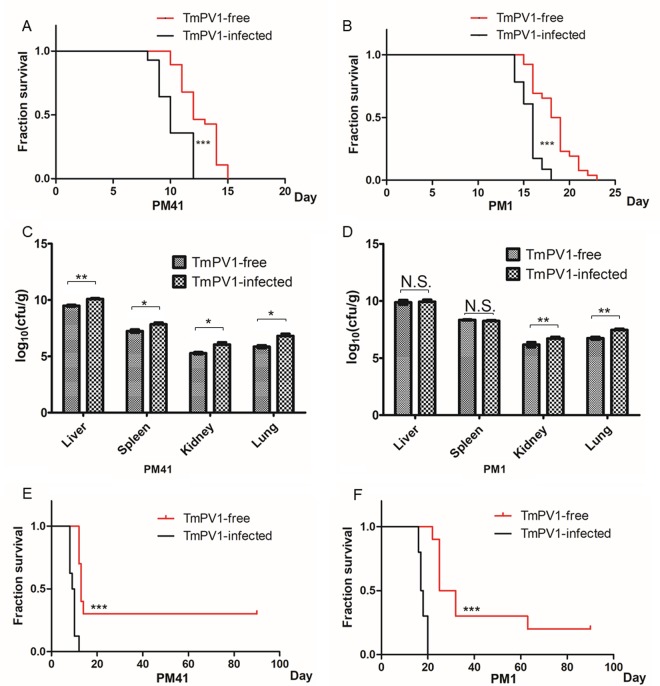
(A and B) Survival of mice challenged with 8 × 10^6^ conidia of TmPV1-free T. marneffei or TmPV1-infected T. marneffei isolate PM41 (A) and PM1 (B). (C and D) Fungal loads in the liver, spleen, kidney, and lung of mice challenged with isolate PM41 (C) and PM1 (D). (E and F) Survival of mice challenged with 4 × 10^6^ conidia of TmPV1-free T. marneffei or TmPV1-infected T. marneffei isolate PM41 (E) and PM1 (F). For survival analysis, groups of 10 BALB/c female mice were challenged intravenously with 8 × 10^6^ conidia and survival was recorded daily. The Kaplan-Meier method and the log-rank test were performed for statistical analysis. ***, *P* < 0.0001. For fungal load analysis, groups of 6 BALB/c female mice were challenged intravenously with 8 × 10^6^ conidia. Mice were sacrificed and necropsies were performed at day 12 (PM1) or day 7 (PM41) postinoculation. Statistical analysis was performed for comparisons between mice inoculated with TmPV1-free spore and TmPV1-infected spore of each organ using Student’s *t* test. *, *P* < 0.05; **, *P* < 0.001; N.S., not significant.

**FIG 4:**
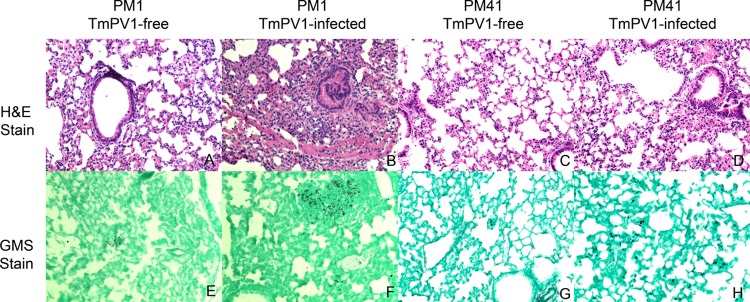
Histopathological sections of lung tissues of mice challenged with TmPV1-infected and TmPV1-free T. marneffei. Formalin-fixed paraffin-embedded lung tissue sections were stained using (A to D) hematoxylin and eosin (H&E) and (E to H) Grocott’s methenamine silver (GMS), respectively (magnification, ×200). More-severe inflammation and higher fungal loads were observed in lungs of mice challenged with TmPV1-infected T. marneffei.

On the other hand, using an established mouse model for T. marneffei (33, 35), BALB/c mice challenged with virus-infected T. marneffei isolates showed a significantly shorter survival time than those challenged with virus-free isogenic isolates at both a lethal dose and a sublethal dose (*P* < 0.0001 for both isolates PM1 and PM41 using the Kaplan-Meier method and the log-rank test) (Fig. 3). In particular, the survival difference was more profound in mice challenged with a sublethal dose of T. marneffei; all mice challenged with TmPV-1-infected T. marneffei died within 20 days, whereas three and two mice challenged with virus-free PM41 and PM1, respectively, were still alive at day 90. The difference between the survival rates of mice infected by the two virus-free T. marneffei isolates was likely due to interisolate variations. While PM41 appeared to be more virulent than PM1 in the present mouse model, both isolates were taken from the blood of patients with systemic penicilliosis (Table S2). For mice challenged with T. marneffei isolate PM41 at a lethal dose, the fungal burden in the liver, spleen, kidneys, and lungs of mice challenged with virus-infected isolate was significantly higher than in those of mice challenged with virus-free isolate at day 7 postchallenge. As for mice challenged with T. marneffei isolate PM1 at a lethal dose, the fungal burden in the kidneys and lungs of mice challenged with virus-infected isolate was also significantly higher than in those of mice challenged with virus-free isolate at day 12 postchallenge (Fig. 3). Histopathological studies also revealed more-severe inflammation and higher fungal loads in organs of mice challenged with virus-infected isolates than in those of mice challenged with virus-free isolates, and the most obvious differences were seen in the lung tissues. The lung tissues of mice challenged with TmPV1-infected isolates showed more marked bronchiolar and alveolar inflammatory cell infiltration and congestion than those of mice challenged with TmPV1-free isolates (Fig. 4). Fungal stains also showed higher fungal loads in the lung tissues of mice challenged with TmPV1-infected isolates. Similar findings were observed in the liver, kidney, and spleen tissues, although the inflammation in those organs was less marked than that observed in lung tissues (Fig. S6).

10.1128/mBio.00947-18.6 FIG S6  Histopathological sections of (A to H) liver, (I to P) kidney, and (Q to X) spleen tissues of mice challenged with TmPV1-infected and TmPV1-free T. marneffei. Formalin-fixed paraffin-embedded liver, kidney, and spleen tissue sections were stained by hematoxylin and eosin (H&E) (panels A to D, I to L, and Q to T) and Grocott’s methenamine silver (GMS) (panels E to H, M to P, and U to X), respectively (magnification, ×200). Download FIG S6, TIF file, 31.3 MB. Copyright © 2018 Lau et al. 2018 Lau et al. This content is distributed under the terms of the Creative Commons Attribution 4.0 International license.

10.1128/mBio.00947-18.8 TABLE S2  Characteristics of the 55 Talaromyces marneffei isolates included in this study. Download TABLE S2, DOCX file, 0.02 MB. Copyright © 2018 Lau et al. 2018 Lau et al. This content is distributed under the terms of the Creative Commons Attribution 4.0 International license.

### Transcriptional changes in TmPV1-infected T. marneffei.

**FIG 5:**
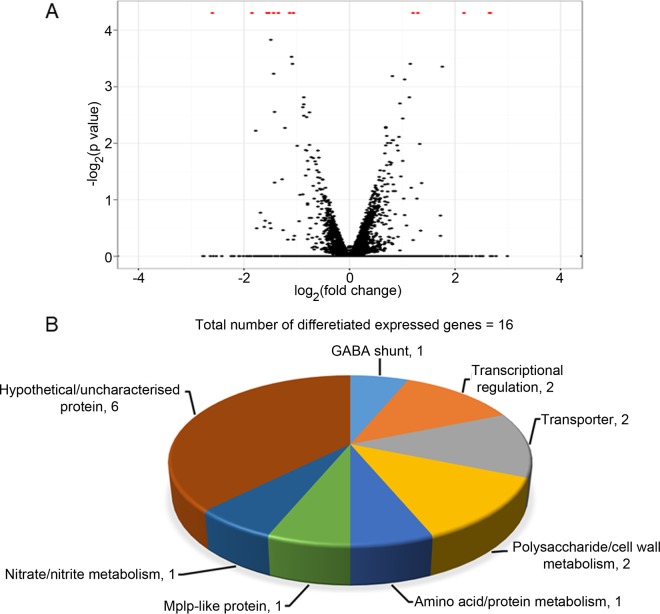
(A) Volcano plot of the RNA-seq data. (B) Classification of differentially expressed genes in TmPV1-infected T. marneffei isolate PM1 compared to isogenic TmPV1-free isolate. A total 16 genes were differentially expressed as indicated by red dots in the volcano plot, among which 11 genes were upregulated and 5 genes were downregulated. Classification of genes was based on the gene ontology terms as determined by UniProt and manual annotation. The number of genes is denoted next to each category designation.

Transcriptome sequencing (RNA-seq) analysis of virus-infected and virus-free T. marneffei PM1 isolates in the yeast phase was performed to identify T. marneffei genes with transcriptional changes after TmPV1 infection. The T. marneffei PM1 isolate was chosen for transcriptomics analysis because sequence data from the T. marneffei PM1 reference genome were available for mapping (34, 45). For each sample, approximately 64 million 100-bp paired-end reads for each sample were obtained and 3.5 Gb of reads were mapped, which represents ~158× coverage of T. marneffei PM1 transcriptome, assuming that 80% of the genome sequences were transcribed. In comparisons between TmPV1-infected and virus-free PM1 isolates, a total of 16 genes were found to be differentially expressed (false-discovery rate [FDR] of ≤0.05), among which 11 genes were upregulated and 5 genes were downregulated in the TmPV1-infected isolate (Fig. 5). These included genes involved in various biological and metabolic processes, such as transcriptional regulation; transporter systems; and polysaccharide, amino acid, and lipid metabolism. The predicted functions of the differentially expressed genes, as determined by manual annotation and gene ontology terms, are summarized in Table S3.

10.1128/mBio.00947-18.9 TABLE S3  Transcriptional changes and predicted function of the 16 differentially expressed genes in TmPV1-infected T. marneffei PM1. Download TABLE S3, DOCX file, 0.02 MB. Copyright © 2018 Lau et al. 2018 Lau et al. This content is distributed under the terms of the Creative Commons Attribution 4.0 International license.

**FIG 6:**
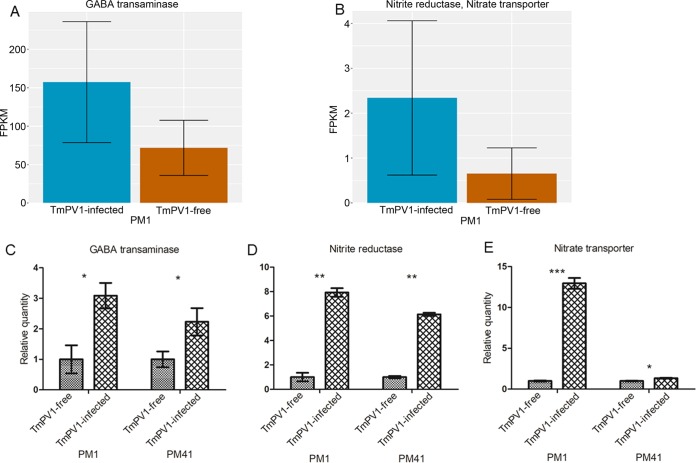
mRNA levels of three differentially expressed genes in TmPV1-free and TmPV1-infected T. marneffei isolates PM1 and PM41 in the yeast phase. (A and B) Relative mRNA levels in fragments per kilobase of transcript per million fragments mapped (FPKM) obtained from RNA-seq data for the GABA transaminase gene (A) and for the nitrite reductase and nitrate transporter fusion genes (B) in isolate PM1. (C to E) Relative mRNA expression levels of GABA transaminase (C), nitrite reductase (D), and nitrate transporter (E) in isolates PM1 and PM41 obtained by qRT-PCR with four independent experimental replicates. Statistical analysis was performed using Student’s *t* test for comparisons between TmPV1-free and TmPV1-infected isogenic isolates. *, *P* < 0.05; **, *P* < 0.001; ***, *P* < 0.0001. Error bars indicate standard errors of the means.

qRT-PCR performed using specific primers targeted to three selected differentially expressed genes, the γ-aminobutyric acid (GABA) transaminase, nitrite reductase, and nitrate transporter genes, confirmed that mRNA expression of these three genes was upregulated in TmPV1-infected T. marneffei PM1 and PM41 isolates compared to the virus-free isogenic isolates (Fig. 6). These three genes were selected for further analysis because they may represent potential virulence factors (46–52).

**FIG 7:**
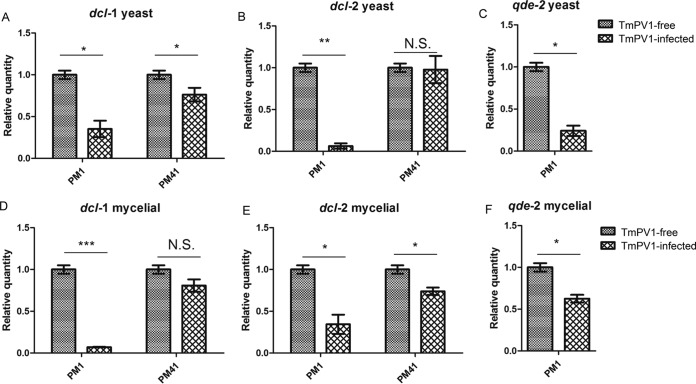
Relative mRNA expression levels of *dcl-1* (A), *dcl-2* (B), and *qde-2* (C) in TmPV1-free and TmPV1-infected T. marneffei isolate PM1 and PM41 in the yeast phase and of *dcl-1* (D), *dcl-2* (E), and *qde-2* (F) in the mycelial phase. Results were obtained from four independent experimental replicates. Statistical analysis was performed using Student’s *t* test for comparisons between TmPV1-free and TmPV1-infected isogenic isolates. *, *P* < 0.05; **, *P* < 0.001; ***, *P* < 0.0001; N.S., not significant. Error bars indicate standard errors of the means.

Since mycoviruses may suppress the RNAi machinery of their fungal hosts, which may serve as an antiviral mechanism (53–55), we also determined the transcriptional changes of the RNAi-related genes in T. marneffei. The mRNA expression of RNAi-related genes, namely, the dicer-1-like gene (*dcl-1*), *dcl-2*, and *qde-2*, was suppressed in TmPV1-infected T. marneffei isolate PM1, in both the yeast phase and mycelial phase, compared to the virus-free isolate (Fig. 7). For isolate PM41, which harbors only *dcl-1* and *dcl-2* and not *qde-2*, the mRNA expression of *dcl-1* and *dcl-2* was also suppressed in the yeast phase and mycelial phase, respectively, in the TmPV1-infected isolate compared to the virus-free isolate (Fig. 7).

## DISCUSSION

This report describes the first discovery of a mycovirus in thermally dimorphic fungi. This novel mycovirus, TmPV1, was phylogenetically most closely related to members of the genus *Gammapartitivirus* of the family *Partitiviridae*, which currently comprises four other genera, namely, *Alphapartitivirus*, *Betapartitivirus*, *Cryspovirus*, and *Deltapartitivirus* (38). While partitiviruses have been found in plants, protists, and fungi, including human-pathogenic fungi such as A. fumigatus, their existence and potential role in dimorphic fungi were unknown (56, 57). In this study, TmPV1 was detected in 12.7% of T. marneffei isolates, with the viral particles further confirmed by transmission electron microscopy, resistance to DNase and RNase, and RT-PCR analysis. TmPV1 possessed 73.3% and 55.9% amino acid identities to PsV-S in the RdRp and capsid proteins, respectively. According to the International Committee on Taxonomy of Viruses (ICTV) species demarcation criteria for classification of members of *Gammapartitivirus*, a novel species is defined as showing ≤90% amino acid identity in the RdRp and/or ≤80% amino acid identity in the capsid protein to the corresponding proteins of members of the genus (http://ictvonline.org/). Therefore, TmPV1 should be classified as a novel species in the genus *Gammapartitivirus*. On the other hand, the RdRp and capsid protein sequences of TmPV1 showed 98.0% to 99.6% and 99.3% to 100% amino acid identities among the seven T. marneffei isolates, suggesting a single viral species. On the basis of the present results, we propose the identification of a novel partitivirus species, TmPV1, in the genus *Gammapartitivirus* under *Partitiviridae*, identified from T. marneffei.

T. marneffei is likely the specific fungal host of TmPV1, which may be transmitted from virus-infected to virus-free isolates. T. marneffei is phylogenetically closely related to other *Penicillium* species and is more closely related to filamentous fungi such as *Aspergillus* than to yeasts (34, 58). Since TmPV1 is closely related to the genus *Gammapartitivirus* in other *Penicillium* and *Aspergillus* species, these partitiviruses probably coevolved with their hosts during their species evolution. In fact, while partitiviruses are widely distributed in plants, protists, and various phyla of fungi, including Ascomycota and Basidiomycota, they have not been reported in yeasts. Further studies should be performed to explore mycoviruses in other dimorphic fungi or yeasts. Mycoviruses, including partitiviruses, are believed to have an obligatory intracellular life cycle without an extracellular phase in nature and to require cell-to-cell contacts for transmission between different host individuals (59). Transmission of mycoviruses even within the same fungal species is often restricted by vegetative incompatibility, which is important for maintaining the integrity of individuals (60–63). Nevertheless, transmission of mycoviruses between vegetatively incompatible isolates of the plant-pathogenic fungus Rosellinia necatrix through anastomosis has been demonstrated *in vitro* (64). Moreover, mycoviruses may be transmitted extracellularly in the laboratory (65, 66). TmPV1 was also successfully transmitted *in vitro* through protoplast transfection to two different virus-free T. marneffei isolates, suggesting that TmPV1 is potentially transmissible among T. marneffei isolates. Further studies may help to reveal the mechanism of viral transmission and possible host factors for viral infection in T. marneffei.

The detection of TmPV1 in both the mycelial and yeast phases of T. marneffei suggests that this mycovirus replicates in different growth phases of the dimorphic fungus. Moreover, the higher viral load detected in the yeast phase should prompt further studies to determine if the virus may replicate more efficiently in yeast cells of T. marneffei. Previous studies have demonstrated the integration of partitivirus and *Totivirus* genes in the nuclear genomes of their hosts (67). Nevertheless, the localization of mycoviruses in infected fungal cells remains largely unknown. More investigations are required to understand the cellular localization and replication cycle of mycoviruses in different stages or forms of their fungal hosts.

The present study represents the first to demonstrate that mycoviruses may enhance the virulence of human-pathogenic fungi in a mammalian model. While most partitiviruses are believed to be cryptic viruses with unknown function, some partitiviruses may exert influence on their fungal hosts with alteration of fungal virulence and growth. For example, partitiviruses isolated from the phytopathogenic fungi Botrytis cinerea and Sclerotinia sclerotiorum were shown to cause hypovirulence in their hosts (68, 69). In another phytopathogenic fungus, Colletotrichum acutatum, curing of a *Gammapartitivirus* was found to reduce production of conidia in its host (70). In the human-pathogenic fungus A. fumigatus, transfection of virus-free isolates with a purified partitivirus has resulted in significant aberrant phenotypic alterations and attenuation of fungal growth (3). Using a moth larvae model, mycovirus A76 was shown to shorten the survival time with higher fungal loads when challenged by virus-infected A. fumigatus compared to the virus-free isogenic isolate (15). These reports suggest that partitiviruses have the ability to influence the biology of their fungal hosts, including human-pathogenic fungi. In this study, we showed that TmPV1-infected T. marneffei was more virulent than virus-free isogenic isolates in mice, with shortened survival time and higher fungal loads in infected organs. Highly reproducible, similar results were obtained using two different T. marneffei isolates transfected with TmPV1. Moreover, the survival difference between virus-free and virus-infected isolates was even more obvious when mice were infected with a lower sublethal dose of T. marneffei. TmPV1-infected T. marneffei causes inflammation in organs of mice, especially the lungs, that is more severe than that seen with virus-free isogenic isolates. Our data suggest that TmPV1 helps T. marneffei to cause more-severe disease and higher mortality in infected hosts, resulting from both higher fungal loads and an exuberant immunopathological response in infected organs. It would be interesting to conduct clinical studies to compare the levels of disease severity of penicilliosis caused by virus-infected and virus-free isolates.

Results of transcriptomics analysis suggest that TmPV1 enhances the virulence of T. marneffei partly through alteration of its gene expression profiles. Compared to the virus-free isolate, TmPV1-infected isolate PM1 showed altered mRNA expression of diverse genes involved in various biological and metabolic processes. Among the 11 upregulated genes, 3 genes, encoding GABA transaminase, nitrate transporter, and nitrite reductase, were reported to play a role in the virulence of various fungi (46–52). GABA transaminase is involved in the GABA shunt, which is involved in hypoxia tolerance and virulence in various fungal species. In Paracoccidioides brasiliensis, GABA shunt-related genes were upregulated under conditions of hypoxia (50). In Fusarium graminearum, a GABA transaminase knockout mutant showed reduced virulence in wheat (49). As for nitrite reductase and nitrate transporter, they are part of the denitrification pathway and involved in nitric oxide (NO) production in various organisms, including fungi. NO is an important signaling molecule for different pathways in bacteria and fungi, including mycotoxin production and induction of necrosis of host cells (46–48, 51, 52). Notably, the nitrite reductase and nitrate transporter genes are located in the same gene cluster in T. marneffei, similarly to A. fumigatus (71). Since knowledge of the potential function of these genes or pathways in T. marneffei remains elusive, further studies are required to determine if the present transcriptional changes may explain the enhanced virulence in TmPV1-infected T. marneffei in mice. In particular, gene knockout studies in TmPV1-infected and virus-free isolates may help researchers understand how the virus may manipulate the fungal virulence and may elucidate the mechanism of virulence enhancement of T. marneffei by this novel mycovirus. In leishmaniasis, it has been shown than a *Leishmania* RNA virus can be recognized by the host Toll-like receptor 3 (TLR3) to subvert the host immune response and promote parasite persistence leishmaniasis in mice (72). In contrast, TmPV1 causes more-severe immunopathological changes in mice infected by T. marneffei. Further mechanistic studies are required to ascertain the mechanism of TmPV1-enhanced fungal virulence.

The altered expression of RNAi-related genes in TmPV1-infected T. marneffei may suggest interplay between the virus and RNAi machinery. In eukaryotic organisms, one of the functions of RNAi is defense against viral infection (73–75). For example, an argonaute-like protein, *agl2*, is required for antiviral defense against a mycovirus in Cryphonectria parasitica (74). A partitivirus infecting R. necatrix induced a higher degree of hypovirulence in a dicer-2-like gene (*dcl-2*) knockout isolate than in the isolate with an intact *dcl-2* gene (75). Mycoviruses have been shown to be targets and suppressors of RNA silencing, with the generation of virus-derived, small interfering RNAs (53). A virulence-attenuating mycovirus, Cryphonectria hypovirus 1, encodes a papain-like p29 protease. Overexpression of this p29 proteinase suppresses the RNAi machinery in its fungal host (54). Small RNA-targeting dsRNA mycoviruses have been discovered in virus-infected Magnaporthe oryzae (76). As a counterdefense system, some mycoviruses, such as those that infect *Aspergillus*, are capable of suppressing the host’s RNAi system (53–55). In a recent study, virus-derived sRNAs were detected in mycovirus-infected A. fumigatus. The microRNA-like RNA and siRNA expression profiles in A. fumigatus were also altered in the presence of mycovirus infection, suggesting that virus-derived sRNAs may influence host mRNA expression (77). Recently, we have discovered microRNA-like RNAs (milRNAs) and RNAi-related genes in T. marneffei which may play a role in posttranscriptional gene regulation (31). Here we showed that TmPV1 may suppress the RNAi machinery of T. marneffei, which may help the virus counteract the potential antiviral effects of RNAi-related genes. In particular, *dcl-1* and *dcl-2* may be more important than *qde-2* in the virus-fungus interaction, since *qde-2* is absent in PM41. Further studies are needed to explore the potential role of the RNAi machinery in antiviral defense for T. marneffei.

## MATERIALS AND METHODS

### T. marneffei isolates and culture conditions.

A total of 55 T. marneffei isolates, isolated from patients with culture-documented penicilliosis, were included (see Table S2 in the supplemental material) (78). T. marneffei isolates were grown on Sabouraud dextrose agar (SDA) (Oxoid, Cambridge, United Kingdom) at 37°C for yeast cultures and at 25°C for mycelial cultures for 7 days as described previously (31, 33). Yeast cells and conidia were collected by scraping and resuspension in 0.1% Tween 20–phosphate-buffered saline (PBS) followed by three washes in PBS before use. For preparation of liquid cultures, cells were resuspended in MilliQ water to obtain a concentration with a McFarland standard of 1.0. One hundred microliters of this inoculum was added to 10 ml yeast extract-peptone-dextrose (YPD) broth (Sigma-Aldrich, St. Louis, MO, USA) for incubation at 37°C for the yeast phase and at 25°C for the mycelial phase for 7 days, with shaking at 250 rpm. All cultures were prepared in triplicate.

### dsRNA isolation and purification.

To isolate dsRNA, 10 ml cultures of the 55 T. marneffei isolates in the mycelial phase were filtered using a Corning bottle top vacuum filter (150 ml) with a pore size of 0.22 µm and a polyethersulfone (PES) membrane (Corning, Corning, NY), and the mycelial mass was collected and subjected to total RNA extraction using RiboPure yeast (Ambion, USA) as described previously (31, 33) followed by treatment with 8 units of DNase I (Ambion)–5 µg/µl RNase A (Qiagen, Hilden, Germany)–2× SSC buffer (Ambion; 1× SSC buffer is 0.3 M NaCl, 30 mM sodium citrate) for 1 h at 37°C, separated by electrophoresis through 1% agarose gels, and visualized under UV light after staining with ethidium bromide, as described previously (79, 80). The isolated dsRNA was purified using a PureLink RNA minikit (Ambion) and stored at −80°C before use.

### Molecular cloning, sequencing, and sequence analysis.

The genome sequences of TmPV1 from the seven T. marneffei isolates were determined using a combination of single-primer amplification techniques and PCR cloning as described previously (81). RNA ligase-mediated rapid amplification of cDNA ends (RLM-RACE) was used to determine the 5′- and 3′-terminal sequences (14, 82). cDNA cloning and sequencing of dsRNA isolated from T. marneffei were performed using a Zero Blunt TOPO PCR Cloning kit (Invitrogen, Carlsbad, CA) as described previously (81). Purified dsRNA was ligated to the phosphorylated 5′-end oligonucleotide 5′-GAGTACAGGTCCGCTCGAATTCTTT-(NH2)-3′ using T4 RNA ligase I (New England Biolabs, Ipswich, MA) with 10% dimethyl sulfoxide (DMSO) at 4°C for 18 h. The oligonucleotide-ligated dsRNA was reverse transcribed using SuperScript III reverse transcriptase (Invitrogen, San Diego, CA) with a specifically designed primer, 5′-AAAGAATTCGAGCGGACCTGTACTC-3′, complementary to the ligated oligonucleotide. Briefly, 200 ng dsRNA was mixed with 0.5 µM primers, and diethyl pyrocarbonate (DEPC)-treated distilled H_2_O was added to reach a final volume of 22.5 µl. The mixture was heated to 95°C for 5 min and chilled on ice for 1 min. The dsRNA was reverse transcribed in a reaction mixture containing 1,000 units of SuperScript III reverse transcriptase (Invitrogen), 50 mM Tris-HCl (pH 8.3 at room temperature), 75 mM KCl, 3 mM MgCl_2_, 5 mM dithiothreitol (DTT), 0.5 mM deoxynucleoside triphosphates (dNTPs), and 100 units of RNaseOut recombinant RNase inhibitor (Invitrogen) and DEPC-treated distilled water to reach a final volume of 50 µl. The mixture was incubated at 25°C for 5 min followed by 55°C for 1 h. After reverse transcription (RT), the mixture was heated at 70°C for 15 min for inactivation of reverse transcriptase. To fill in 3′ ends, the RT products were mixed with 10 mM Tris-HCl (pH 8.3), 50 mM KCl, 1.5 mM MgCl_2_, 0.12 mM dNTPs, and 2.5 units of heat-activated AmpliTaq Gold DNA polymerase (Applied Biosystems, Foster City, CA) and incubated at 72°C for 10 min. The resultant cDNAs were then used for PCR amplification performed with iProof High-Fidelity DNA polymerase (Bio-Rad, Hercules, CA) and the same primer used for RT. Briefly, the cDNA was mixed with 1× iProof PCR buffer, 2 mM MgCl_2_, 0.2 mM dNTPs, 1 µM primers, 1 unit of iProof DNA polymerase, and DEPC-treated distilled water to reach a final volume of 50 µl. DNA amplifications were carried out using an Applied Biosystems Veriti 96-Well Fast Thermal Cycler (Applied Biosystems). The mixture was incubated at 98°C for 5 min for denaturation and enzyme activation and amplified in 30 cycles of 98°C for 30 s, 68°C for 30 s, and 72°C for 1 min 30 s followed by a final extension of 72°C for 10 min. The amplified PCR products were cloned into pCR-Blunt II-TOPO vector provided in a Zero Blunt TOPO PCR Cloning kit (Invitrogen) according to the manufacturer’s instructions and then transformed into competent cells of Escherichia coli DH5α by electroporation using a Gene Pulser (Bio-Rad) and a Gene Pulser cuvette with 0.2-cm width, under the following conditions: voltage, 2.5 kV; capacitance, 25 μF; resistance, 200 Ω. Clones were selected on Luria-Bertani agar (Becton, Dickinson and Company, Franklins Lakes, NJ, USA) supplemented with 50 µg/ml kanamycin, 12.5 µM IPTG (isopropyl-β-d-thiogalactopyranoside), and 1.25 mg X-Gal (5-bromo-4-chloro-3-indolyl-β-d-galactopyranoside) (Promega, Madison, WI). Positive clones were sequenced and analyzed with BLASTX on the NCBI website (http://blast.ncbi.nlm.nih.gov/Blast.cgi).

Gaps in sequences between different clones were determined using RT-PCR amplification and specifically designed primers based on obtained sequences. To obtain the 5′- and 3′-terminal sequences, the 3′ terminus of each strand of dsRNA was ligated to an adenylated 3′ adapter using a poly(A) tailing kit (Ambion) according to the manufacturer’s instructions (31). The polyadenylated dsRNA was then denatured and used for RT with SuperScript III reverse transcriptase (Invitrogen), 0.5 µM specific primers (5′-ATACATACCAACCGGCCCTTC-3′ and 5′-GAATCCAGCGTTTCATCACTGCC-3′ for 5′ and 3′ ends of dsRNA-1, respectively, and 5′-CCCAAAGAAGGTATAGGCATCTC-3′ and 5′-CCCGACCGACGAGTCATTGTG-3′ for 5′ and 3′ ends of dsRNA-2, respectively) and 0.5 µM oligo(dT) with anchor sequence 5′-GACCACGCGTATCGATGTCGACTTTTTTTTTTTTTTTTV-3′. The cDNAs were amplified by seminested PCR techniques using 64 nM concentrations of the specific primers used for RT, a 1 µM concentration of a primer specific to the oligo(dT) anchor sequence (5′-GACCACGCGTATCGATGTCGAC-3′), and inner primers (5′-CGTACACTTGTCGATCGAATTC-3′ and 5′-CTCATTGGATTGTGGATTGGTGG-3′ for 5′ and 3′ ends of dsRNA-1, respectively, and 5′-TCACCTTGGGGCAGACCCATA-3′ and 5′-CTGAACGTTTCCCAGCGTGCA-3′ for 5′ and 3′ ends of dsRNA-2, respectively). The PCR products were subjected to gel purification using a QIAquick gel purification kit (Qiagen, Hilden, Germany). Both strands of the PCR products were sequenced twice with an ABI Prism 3700 DNA analyzer (Applied Biosystems) using the PCR primers. Sequence-specific primers were used to confirm the final genome sequences, and every base was determined by sequencing overlapping clones or PCR products in both orientations.

Sequence analysis, alignments, and phylogenetic analysis were carried out by using CLUSTAL_W in MEGA version 6 (83), BLAST, and NCBI ORF Finder software (https://www.ncbi.nlm.nih.gov/gorf). The sequences of previously reported mycoviruses were retrieved from the NCBI GenBank database and used for comparative analyses. Phylogenetic trees were constructed by the maximum likelihood method based on the best predicted model, the Le_Gascuel_2008 model with gamma distributions, for RdRp and the General Reverse Transcriptase with Frequency model, gamma distributions, for capsid protein, using MEGA version 6 (83). Pairwise comparison of the predicted amino acid sequences of RdRp and capsid protein between TmPV1 and other members of *Partitiviridae* were performed using LaserGene software (41).

### Mycovirus detection in different T. marneffei isolates and growth phases.

To confirm the absence of TmPV1 from the other 48 T. marneffei isolates which did not show dsRNA bands on gel electrophoresis, RT-PCR was performed using purified RNA extracted from their mycelial mass and specific primers (5′-CGAGAGGAGCCAGCTATGAC-3′ and 5′-CGACGACCCTTCCTTCCTTT-3′) designed to target a 484-bp region of TmPV1 dsRNA-1. cDNA was prepared from purified RNA as described above, using 0.5 µM concentrations of each of the RT primers (5′-TCTGATCCCCCATCGGTTGA-3′ and 5′-CCAGTGCACCACTCAGTGTA-3′). cDNA was mixed with 10 mM Tris-HCl (pH 8.3), 50 mM KCl, 2.5 mM MgCl_2_, 0.2 mM dNTPs, 1 µM PCR primers, 0.625 units of AmpliTaq Gold DNA polymerase (Applied Biosystems), and DEPC-treated distilled water to reach a final volume of 25 µl. DNA amplifications were carried out using an Applied Biosystems Veriti 96-Well Fast Thermal Cycler (Applied Biosystems). The mixture was incubated at 95°C for 5 min for denaturation and enzyme activation and amplified in 40 cycles of 95°C for 30 s, 60°C for 30 s, and 72°C for 1 min followed by a final extension of 72°C for 10 min. The PCR product was separated by gel electrophoresis and visualized under UV light.

To detect the presence of TmPV1 in different growth phases, dsRNAs were also extracted and purified from the yeast forms of the seven T. marneffei isolates with dsRNA bands in mycelial forms observed in gel electrophoresis, using extraction methods described above. The purified dsRNAs were separated by gel electrophoresis and visualized under UV light.

### Purification of viral particles and transmission electron microscopy.

Viral particles were purified from mycelial cultures of T. marneffei isolate PM40 found to contain dsRNA elements. Purification of viral particles was performed as described previously (84). Briefly, T. marneffei isolate PM40 was grown at 25°C in YPD broth for 7 days until the stationary phase was reached. The culture was filtered, and the mycelial mass was homogenized with 425 to 600 µm acid-washed glass beads (Sigma) by vortex mixing in ice-cold buffer A (50 mM Tris, 150 mM NaCl, 5 mM EDTA, 1 mM DTT, pH 7.6). One volume of chloroform was then added, and the mixture was homogenized and centrifuged at 5,200 × *g* for 20 min. The aqueous layer was subjected to two cycles of differential centrifugation (144,400 × *g* for 150 min and 12,000 × *g* for 10 min). The resultant pellet was resuspended in buffer A. The final purification employed rate-zonal centrifugation in sucrose gradients (10% to 50%). A gradient was made in buffer A and centrifuged at 103,600 × *g* for 150 to 160 min. The major band containing the viral particles was withdrawn with a syringe from the side of the tube. Viral particles were concentrated by overnight centrifugation at 144,400 × *g*. The pellet was resuspended in buffer A–50% glycerol. All centrifugation steps were performed at 4°C. A drop of purified virus suspension was placed on a Formvar/carbon-coated copper grid and stained negatively using 1% uranyl acetate and examined for viral particles using a Philips CM-100 transmission electron microscope (TEM) (Philips, Amsterdam, Netherlands).

### Quantitative RT-PCR for comparison of viral loads between the mycelial and yeast phases.

Mycelial and yeast liquid cultures of the seven T. marneffei isolates infected with TmPV1 were performed as described above. Total RNAs were extracted from the 7-day cultures using RiboPure Yeast (Ambion, USA) as described above. Reverse transcription was performed using SuperScript III reverse transcriptase (Invitrogen) with 12.5 nM random primer (Invitrogen) and the conditions stated above, with the incubation temperature for reverse transcriptase at 50°C. Real-time PCR assays were performed as described previously (31), using the actin gene for normalization (5′-GAACGTGAAATCGTCCGT-3′ and 5′-AGCAAGAATGGAACCACC-3′) and the TmPV1 capsid gene (5′-TCGCTTCCTTCTTCGCGATT-3′ and 5′-TCCCAATCACGGTTGTCACC-3′) as the target gene for detection. cDNA was amplified in a LightCycler 2.0 instrument (Roche, Switzerland) in 20-µl reaction mixtures containing FastStart DNA Master SYBR green I. A mix reagent kit (Roche), 8 µl of 10-fold-diluted cDNA, 2 mM MgCl_2_, and 0.5 µM primers were processed at 50°C for 5 min followed by 40 cycles of 95°C for 15 s and 60°C for 1 min and the use of a dissociation curve in SDS 2.4 control software (31). Statistical analyses of the qRT-PCR data were performed using a two-tailed Student’s *t* test (R version 3.1.1) (85).

### Infection of virus-free T. marneffei isolates with TmPV1.

To investigate the biological effects of TmPV1 in T. marneffei, the virus-free isolates (PM1 and PM41) were subjected to virus infection using protoplast transfection of mycelia with purified viral particles from isolate PM40 (3, 86). Protoplasts of virus-free isolates were prepared as described previously (87), with modifications. Briefly, 10 ml of 3-day-old mycelial or yeast cultures were collected, placed in yeast extract-peptone dextrose (YPD) broth, and washed using 0.6 M MgSO_4_. Washed mycelial masses were mixed with 10 mg/ml lysing enzyme from Trichoderma harzianum (Sigma-Aldrich, St. Louis, CA)–1.2 mg/ml bovine serum albumin (BSA)–osmotic buffer (1.2 M MgSO_4_, 10 mM sodium phosphate, pH 5.8) and incubated at 37°C with gentle shaking for 1 h. Protoplasts were collected and mixed with purified viral particles for coincubation at room temperature for 30 min before being spread on SDA with 1.2 M sorbitol as an osmotic stabilizer. Plates were incubated at room temperature for 7 days prior to testing for virus infection. Single colonies were picked for testing of virus infection using the RT-PCR assay with TmPV1-specific primers as described above. Amplification and sequencing of isolate-specific *mp1* genes were used for confirmation of isogenic T. marneffei isolates before and after virus infection (78).

### Effects of TmPV1 infection on mycelial growth rates.

Approximately 500 freshly collected conidia of virus-infected and virus-free T. marneffei isolates PM1 and PM41 were inoculated onto the center of an SDA plate and incubated at room temperature for 7 days. The colony diameters were measured at 48 h followed by measurement performed every 24 h until day 7 or until the colonies reached the edge of the SDA plate (3, 14). Colony morphological differences between virus-infected and virus-free isolates were recorded.

### Scanning electron microscopy.

Scanning electron microscopy for T. marneffei was performed as described previously (88), with modifications. TmPV1-infected and TmPV1-free PM1 and PM41 isogenic isolates in the yeast phase and mycelial phase were cultured on round filter membranes (10 mm in diameter with a pore size of 1 µm) on tap water agar until colonies were observed. The membrane was fixed in 6% (wt/vol) glutaraldehyde for 48 h following dehydration using two changes in absolute ethanol. Dehydrated samples were subjected to critical point drying in a CPD 030 Critical Point Drier (Bal-Tec; Balzers, Liechtenstein) using carbon dioxide as the drying agent. Critical-point-dried samples were mounted onto an aluminum stub and coated with palladium using a SCD 005 Cool Sputter Coater (Bal-Tec). Coated samples were examined in a Hitachi S-3400N variable-pressure scanning electron microscope (Hitachi High-Technologies, Tokyo, Japan).

### Effects of TmPV1 infection on intracellular survival in murine macrophages.

Analyses of intracellular survival in murine macrophage J774 cell cultures (Sigma-Aldrich, St. Louis, MO) were performed as described previously (33). Briefly, J774 cells were seeded onto 24-well tissue culture plates at 4 × 10^5^ cells per well with Dulbecco’s modified Eagle’s medium (Invitrogen). Cells were incubated at 37°C with 5% CO_2_ for 24 h. After replacement with fresh medium, virus-infected and virus-free T. marneffei isolates PM1 and PM41 were added at a multiplicity of infection (MOI) of 1 and incubated for 2 h to allow infection. The monolayers were washed with 240 U/ml nystatin to kill the extracellular conidia and then washed with warm Hanks’ buffered salt solution for nystatin removal before replacement with fresh media for further incubation for 24 h. Cells were lysed with 1% Triton X-100, and cell lysates were diluted and plated on SDA for determination of CFU counts of intracellular T. marneffei. The CFU counts determined from cell lysates after 2 h of infection were considered to represent the initial inocula and used as baseline values, and the CFU counts determined after 24 h of infection were used for calculation of the intracellular survival of T. marneffei in macrophages.

### Ethics statement.

Experiments involving animals were performed in accordance with the NIH guidelines, Animals (Control of Experiments) Ordinance, Hong Kong Law, and Prevention of Cruelty to Animals Ordinance. Animal experiments were approved by the Committee on the Use of Live Animals in Teaching and Research, the University of Hong Kong (project number 3565–15). Animals were euthanized by overdoses of anesthetics.

### Effects of TmPV1 infection on fungal virulence in challenged mice.

The *in vivo* virulence of virus-infected and virus-free T. marneffei PM1 and PM40 isolates was studied using an established mouse model as described previously (33, 35). Briefly, groups of 10 BALB/c (H-2^d^) mice (Laboratory Animal Unit, the University of Hong Kong, Hong Kong) that were 5 to 8 weeks old (weight, 18 to 22 g) were challenged intravenously with 8 × 10^6^ conidia of each T. marneffei isolate (lethal dose for wild-type isolate). To further validate the TmPV1-induced hypervirulence in mice, additional groups of 10 mice were inoculated with a lower inoculum (4 × 10^6^ conidia of TmPV1-free or TmPV1-infected isogenic isolates). The survival of mice was recorded daily for 90 days, and the survival rates were compared using the Kaplan-Meier method and the log-rank test for statistical analysis. All experiments were performed in triplicate. Since statistically significant survival differences were demonstrated between virus-free and virus-infected isolates, additional groups of six mice for each T. marneffei isolate challenged intravenously with 8 × 10^6^ conidia were included and were sacrificed at day 7 for isolate PM41 and day 12 for isolate PM1 (before the end of the expected days of survival). Necropsies of mice were performed to obtain the liver, spleen, lungs, and kidneys for determination of fungal CFU in infected organs. For histopathological studies, organs were fixed in neutral buffered 10% formalin and embedded in paraffin. Paraffin-embedded sections were stained with hematoxylin and eosin (H&E) or Grocott’s methenamine silver (GMS). The stained sections were observed under a light microscope (37).

### RNA-seq analysis of transcriptional changes in TmPV1-infected T. marneffei.

To identify the potential biological role of mycovirus in T. marneffei, the effects of TmPV1 infection were determined at the transcriptional level by transcriptomics studies using RNA-seq analysis (45). Briefly, conidia of virus-infected and virus-free PM1 T. marneffei isolates were cultured in YPD broth at 37°C for 2 days to obtain yeast forms (the pathogenic phase). The total RNAs were extracted using a RiboPure yeast kit (Ambion). To control for global transcriptional differences, the total RNA concentration was adjusted according to the RNA content before the rRNA-depleted RNA-seq analysis was performed. rRNA depletion was carried out using a Ribo-Zero Gold rRNA removal kit (Yeast) according to the instructions of the manufacturer (Illumina, San Diego, CA). RNA was quantified using a Qubit 2.0 fluorometer (Life Technologies, Inc., Grand Island, NY). RNA integrity number (RIN) value determinations and electrophoretogram reads of all RNA samples were performed using a 2100 Bioanalyzer (Aligent Genomics, Santa Clara, CA). Sequencing was performed using a HiSeq 1500 instrument (Illumina). The reads were trimmed on the basis of their qualities by the use of FastQC by Trimmomatic (89). Filtered reads were used to map the T. marneffei PM1 reference genome sequence (NCBI BioProject accession no. PRJNA251717) (45) with Tophat v2.1.0 (90). Cufflinks v2.0.2 software (90) was used to calculate the number of fragments per kilobase of transcript per million fragments mapped (FPKM) (90) and the confidence intervals for each gene. For differential expression analysis, mapped reads were counted and differentially expressed genes were detected by using Cuffdiff in the Cufflinks v2.2.1 package (90). Genes with a FDR of ≤0.05 as reported by Cuffdiff were considered to be differentially expressed. Four independent biological replicates were included in all experiments. Manual annotation was performed on differentially expressed genes based on gene sequence data from the reference genome of T. marneffei isolate PM1 (45), using BLASTX and a conserved-domain search on NCBI. Biological processing and gene classifications were based on the gene ontology terms determined by UniProt (http://www.UniProt.org) and a literature search of protein homologues. The plots for RNA-seq analyses were prepared by the use of the CummeRbund package in the R (version 3.1.1) (85) environment (90).

### qRT-PCR for transcriptional changes of RNAi-related and differentially expressed genes in TmPV1-infected T. marneffei.

mRNA expression of three selected differentially expressed genes, i.e., the GABA transaminase, nitrite reductase, and nitrate transporter genes, in the yeast phase and of RNAi-related genes *dcl-1*, *dcl-2*, and *qde-2* in both the yeast and mycelial phases of virus-infected and virus-free T. marneffei PM1 isolates were determined using qRT-PCR as described previously (Table S4) (31). Briefly, extracted total RNA of T. marneffei isolates was reverse transcribed and cDNA was amplified in real-time PCR using primers and conditions described previously (31) in an ABI 7900HT Fast real-time PCR system. The actin gene was used for normalization. qRT-PCR results were analyzed using SDS 2.4 software, and statistical analysis was performed using Student’s *t* test.

10.1128/mBio.00947-18.10 TABLE S4  Primers used for qRT-PCR analysis of differentially expressed and RNAi-related genes. Download TABLE S4, DOCX file, 0.02 MB. Copyright © 2018 Lau et al. 2018 Lau et al. This content is distributed under the terms of the Creative Commons Attribution 4.0 International license.

### Accession number(s).

The nucleotide sequences of the seven TmPV1 genomes have been deposited in GenBank under accession numbers KM235304 to KM235317. The RNA-seq raw data have been deposited in NCBI under BioProject accession number PRJNA353903.
