# Supplementary figures and images for: Novel Partitivirus Enhances Virulence of and Causes Aberrant Gene Expression in Talaromyces marneffei
Source: mBio. 2018 Jun 12;9(3):e00947-18. doi: 10.1128/mBio.00947-18 (PMC6016240; doi:10.1128/mBio.00947-18)

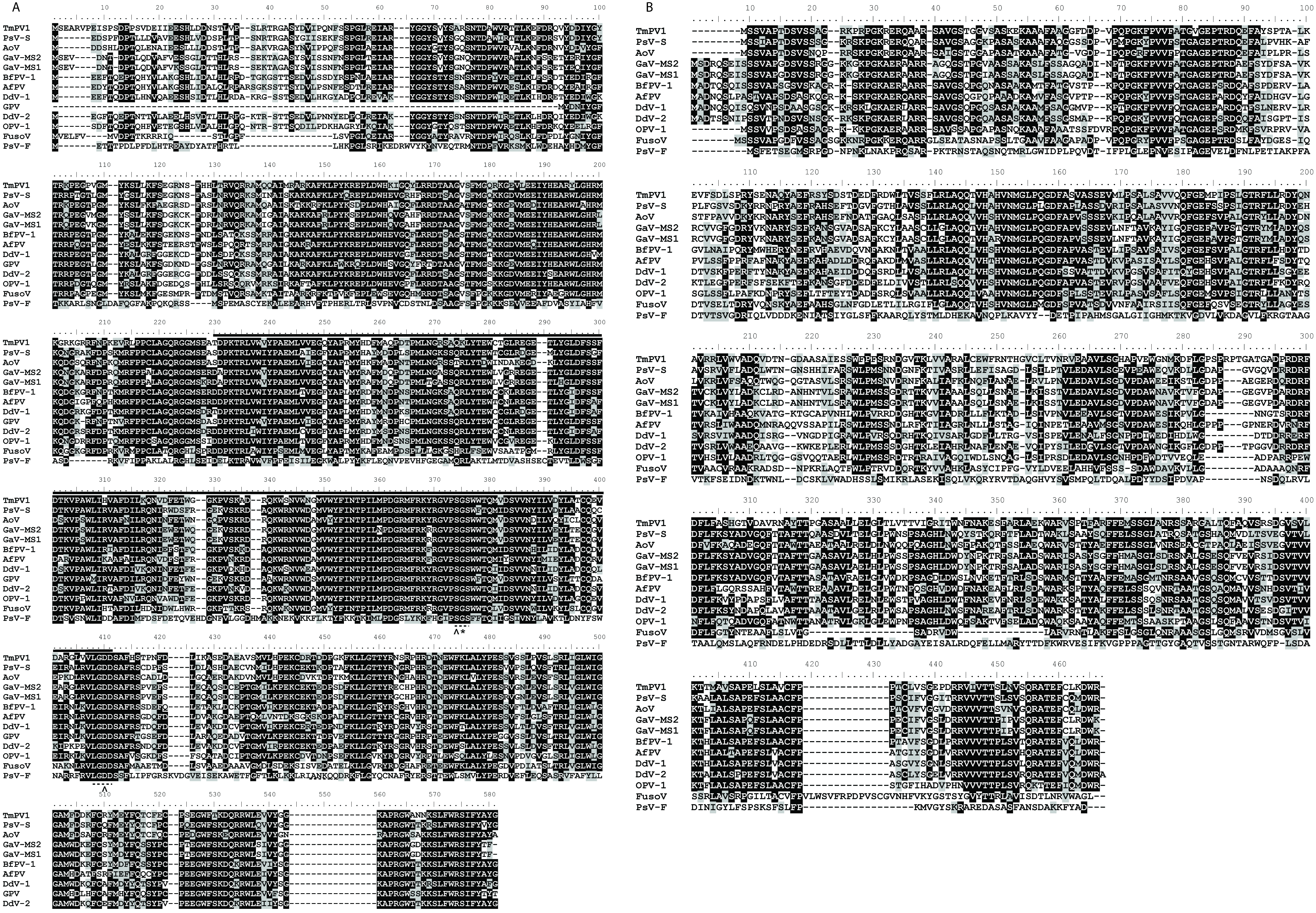

Supplement: FIG S1 [file mbo003183923sf1.tif]

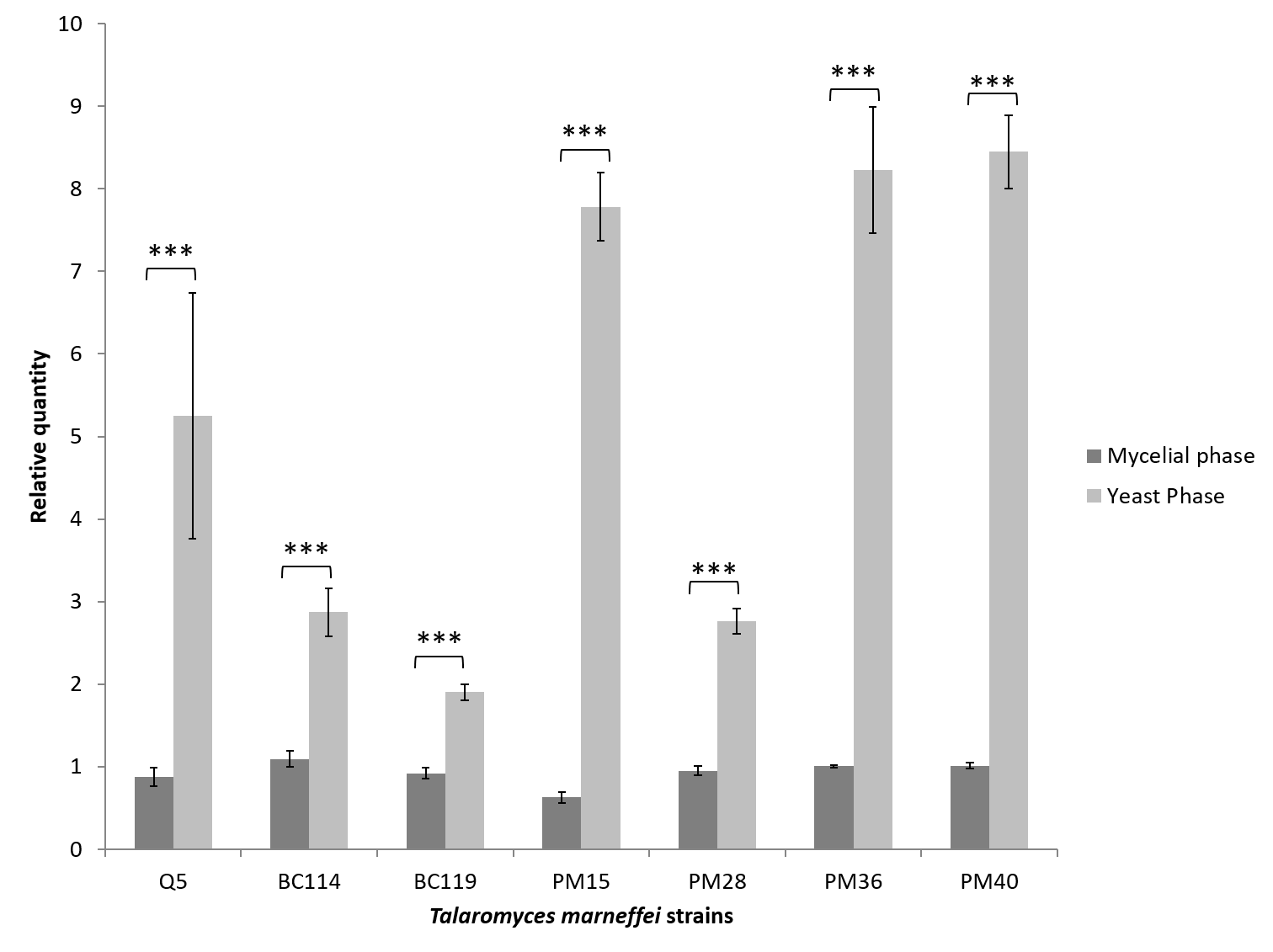

Supplement: FIG S2 [file mbo003183923sf2.tif]

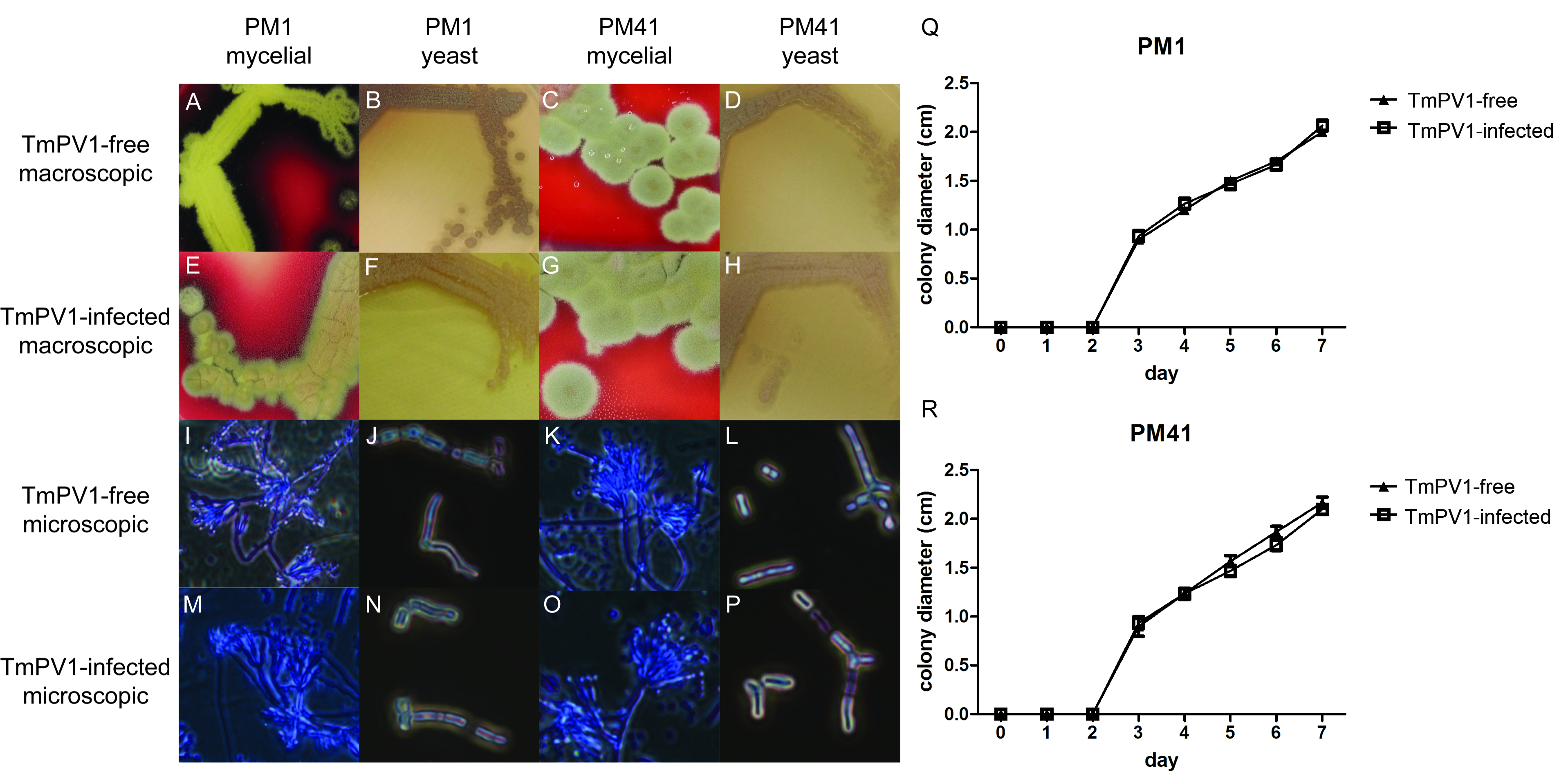

Supplement: FIG S3 [file mbo003183923sf3.tif]

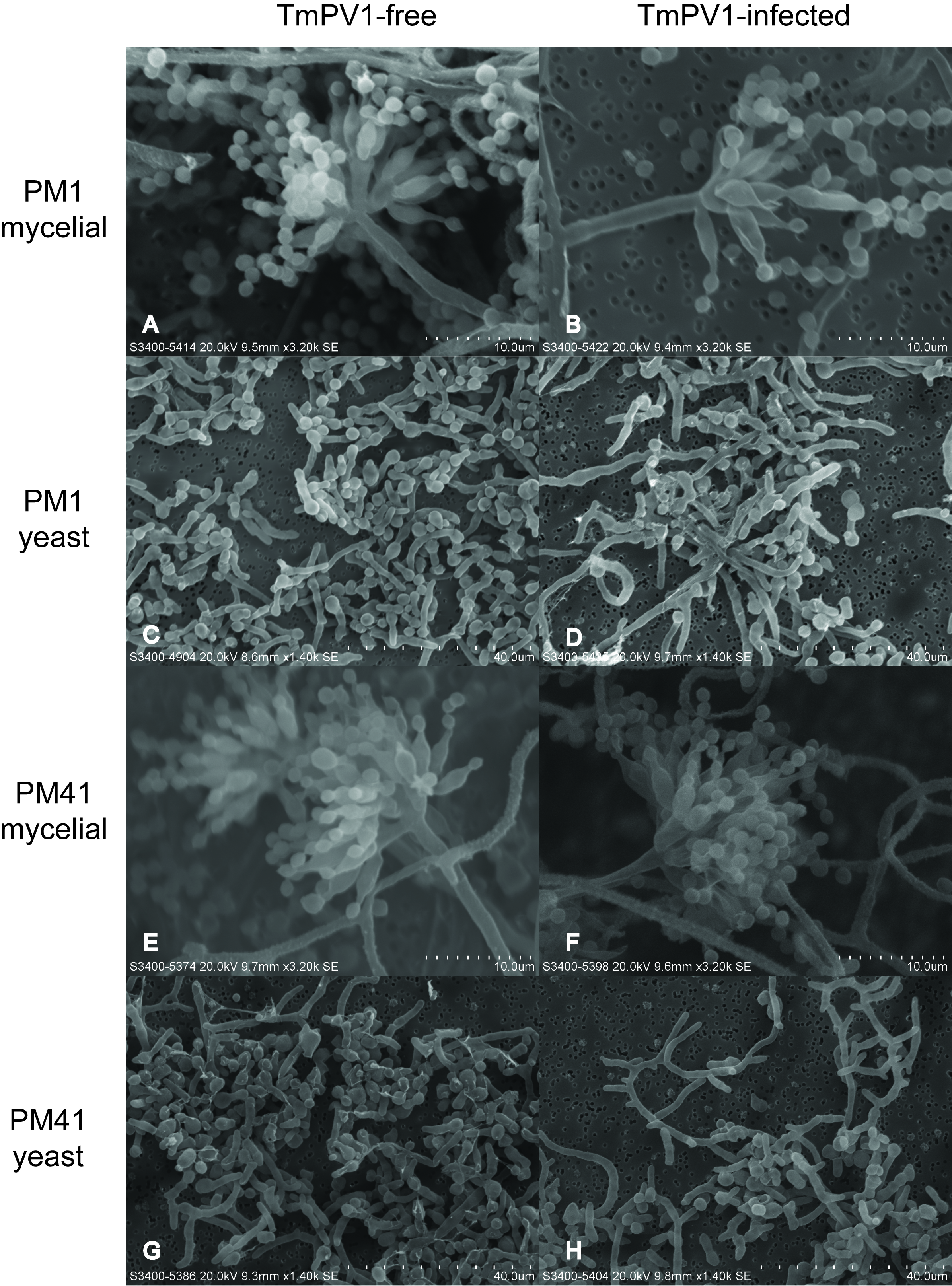

Supplement: FIG S4 [file mbo003183923sf4.tif]

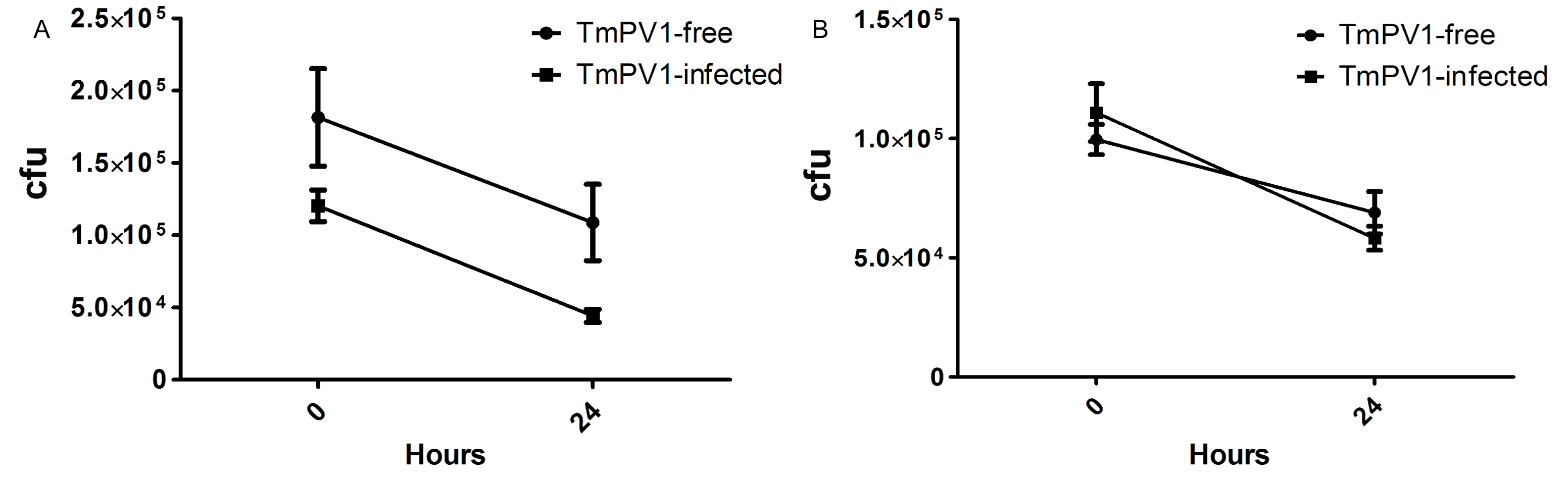

Supplement: FIG S5 [file mbo003183923sf5.tif]

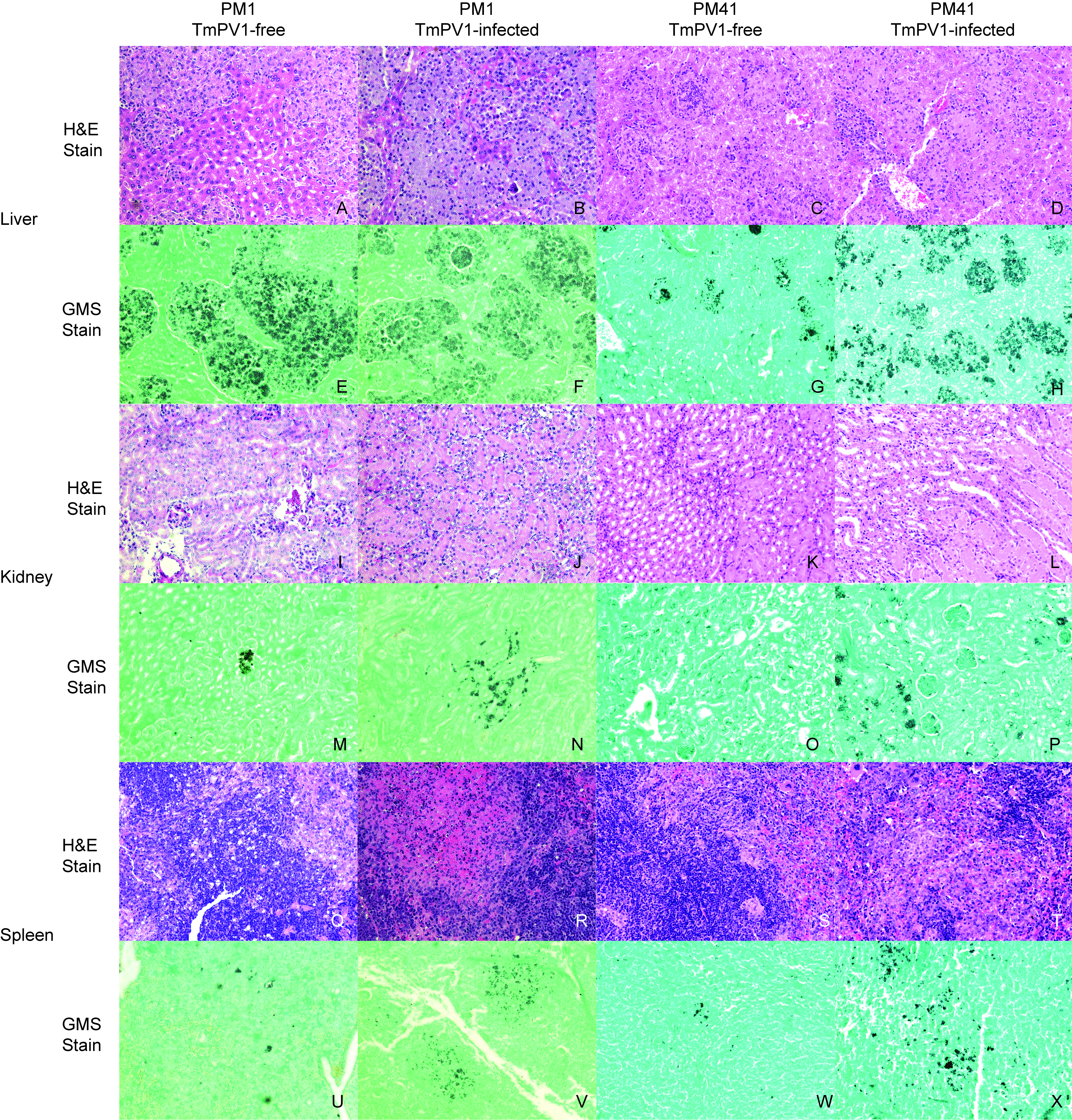

Supplement: FIG S6 [file mbo003183923sf6.tif]
