# Supplementary material for: Novel Partitivirus Enhances Virulence of and Causes Aberrant Gene Expression in Talaromyces marneffei
Source: mBio. 2018 Jun 12;9(3):e00947-18. doi: 10.1128/mBio.00947-18 (PMC6016240; doi:10.1128/mBio.00947-18)
Supplement: TABLE S1 [file mbo003183923st1.docx]

**Table S1. Homology of the predicted amino acid sequence of TmPV1 RdRp and Capsid Protein with corresponding regions of other *Partitiviridae***

| Genus | *Gammapartitivirus* | | | | | | | | | | | |  | Unclassified |
| --- | --- | --- | --- | --- | --- | --- | --- | --- | --- | --- | --- | --- | --- | --- |
|  | TmPV1 | PsV-S* | AoV | GaV-MS2 | GaV-MS1 | BfPV-1 | AfPV | DdV-1 | GPV | DdV-2 | OPV-1 | FusoV | | PsV-F |
|  | Identity^c^ of RdRp amino acid sequence (%) | | | | | | | | | | | | | |
| TmPV1^a^ | --- | 73.3 | 71.8 | 70.7 | 70.1 | 67.0 | 66.0 | 64.9 | 74.0 | 64.2 | 64.0 | 60.3 | | 26.8 |
| PsV-S | 55.9 | --- | 72.0 | 72.9 | 72.3 | 70.5 | 70.1 | 69.9 | 76.4 | 68.5 | 67.2 | 60.5 | | 26.6 |
| AoV | 54.9 | 63.7 | --- | 68.4 | 67.8 | 65.7 | 66.2 | 62.9 | 71.8 | 63.5 | 63.8 | 59.5 | | 25.5 |
| GaV-MS2 | 49.8 | 57.4 | 57.3 | --- | 98.5 | 71.0 | 65.8 | 66.7 | 76.1 | 65.8 | 65.8 | 61.1 | | 28.2 |
| GaV-MS1 | 49.3 | 57.1 | 57.0 | 99.5 | --- | 70.6 | 65.4 | 66.4 | 76.1 | 65.4 | 65.6 | 61.1 | | 28.0 |
| BfPV-1 | 51.4 | 56.0 | 58.2 | 56.9 | 56.7 | --- | 80.6 | 81.4 | 75.1 | 82.2 | 73.7 | 59.2 | | 27.8 |
| AfPV | 50.8 | 53.7 | 55.7 | 50.1 | 49.9 | 63.3 | --- | 74.2 | 71.1 | 75.9 | 69.9 | 59.3 | | 27.4 |
| DdV-1 | 48.2 | 53.6 | 51.2 | 52.3 | 52.1 | 61.8 | 65.1 | --- | 72.4 | 87.6 | 71.8 | 57.0 | | 27.8 |
| GPV | n/a^b^ | n/a | n/a | n/a | n/a | n/a | n/a | n/a | --- | 72.0 | 70.0 | 63.4 | | 29.3 |
| DdV-2 | 50.0 | 55.6 | 57.5 | 54.6 | 54.4 | 62.1 | 63.0 | 64.0 | n/a | --- | 71.4 | 59.3 | | 27.4 |
| OPV-1 | 45.9 | 51.6 | 50.4 | 51.0 | 50.8 | 54.4 | 53.3 | 52.5 | n/a | 51.8 | --- | 57.0 | | 27.2 |
| FusoV | 41.3 | 40.5 | 39.6 | 41.6 | 41.3 | 39.7 | 39.5 | 38.6 | n/a | 39.4 | 36.7 | --- | | 28.2 |
| PsV-F | 12.4 | 16.1 | 15.2 | 15.3 | 15.3 | 13.7 | 13.2 | 15.6 | n/a | 13.2 | 12.4 | 11.7 | | --- |
|  | Identity of Capsid Protein amino acid sequence (%) | | | | | | | | | | | | | |

^a^ TmPV1, Talaromyces marneffei partitivirus 1; PsV-S, Penicillium stoloniferum virus-S; AoV; Aspergillus ochraceus virus; GaV-MS2, Gremmeniella abietina RNA virus-MS2; GaV-MS1, Gremmeniella abietina RNA virus-MS1; BfPV-1, Botryotinia fuckeliana partitivirus-1; AfPV, Aspergillus fumigatus partitivirus-1; DdV-1, Discula destructiva virus-1; GPV, Grapevine partitivirus; DdV-2, Discula destructiva virus-2; OPV-1, Ophiostoma partitivirus-1; FusoV, Mycovirus FusoV; PsV-F, Penicillium stoloniferum virus-F.

^b^ Sequences were not available.

^c^ Value are the identity scores, calculated by Clustal W method with weighted residue table in the LaserGene program.

* Type species of genus *Gammapartitivirus*
