# Supplementary material for: Novel Partitivirus Enhances Virulence of and Causes Aberrant Gene Expression in Talaromyces marneffei
Source: mBio. 2018 Jun 12;9(3):e00947-18. doi: 10.1128/mBio.00947-18 (PMC6016240; doi:10.1128/mBio.00947-18)
Supplement: TABLE S2 [file mbo003183923st2.docx]

**Table S2. Characteristics of the 55 *Talaromyces marneffei* strains included in this study^a^**

| Strain | Year of isolation | Origin | | Patient | | TmPV1 |
| --- | --- | --- | --- | --- | --- | --- |
|  |  | Country | Specimen | Sex/Age (year) | HIV status |  |
| PM1 | N/A | Hong Kong | Blood | M/2 | − | − |
| PM2 | 1994 | Hong Kong | LN biospy | M/34 | + | − |
| PM3 | 1994 | Hong Kong | Blood | M/51 | + | − |
| PM4 | 1993 | Philippines | Blood | F/30 | − | − |
| PM5 | N/A | Hong Kong | BAL fluid | M/23 | − | − |
| PM6 | N/A | Hong Kong | Blood | M/N/A | + | − |
| PM7 | N/A | Hong Kong | Blood | M/N/A | + | − |
| PM8 | N/A | Hong Kong | Blood | M/N/A | + | − |
| PM9 | N/A | Hong Kong | Bone marrow | F/21 | − | − |
| PM10 | N/A | Hong Kong | Bone marrow | M/3 | − | − |
| PM11 | 1998 | Hong Kong | Pleural fluid | F/65 | − | − |
| PM12 | N/A | Hong Kong | Blood | N/A | N/A | − |
| PM13 | 1996 | Hong Kong | Blood | F/45 | − | − |
| PM14 | 1996 | Hong Kong | Blood | M/52 | + | − |
| PM15 | N/A | Hong Kong | Blood | N/A | N/A | + |
| PM16 | N/A | Hong Kong | Blood | N/A | N/A | − |
| PM17 | 1998 | Hong Kong | Sputum | M/60 | − | − |
| PM18 | 1999 | Thailand | Blood | F/22 | N/A | − |
| PM19 | 1999 | Hong Kong | Blood | M/35 | + | − |
| PM20 | 1999 | Thailand | Blood | F/26 | + | − |
| PM21 | 1999 | Hong Kong | Blood | M/39 | + | − |
| PM22 | 1999 | Hong Kong | Blood | M/73 | − | − |
| PM23 | 2000 | Hong Kong | Blood | M/63 | + | − |
| PM24 | 2001 | Hong Kong | Blood | M/39 | + | − |
| PM25 | 2002 | Hong Kong | Blood | M/47 | + | − |
| PM26 | 2003 | Philippines | Blood | F/35 | + | − |
| PM27 | 2004 | Hong Kong | Blood | M/25 | + | − |
| PM28 | 2004 | Hong Kong | Blood | M/57 | − | + |
| PM29 | 2005 | Hong Kong | Blood | F/45 | + | − |
| PM30 | 2005 | Hong Kong | Blood | M/33 | + | − |
| BC114 | N/A | Hong Kong | Skin biopsy | F/38 | − | + |
| PM32 | 2005 | Hong Kong | Blood | F/40 | + | − |
| PM33 | 2005 | Vietnam | Blood | M/22 | + | − |
| PM34 | 2004 | Hong Kong | Blood | M/48 | + | − |
| BC119 | 2005 | Hong Kong | Blood | M/41 | N/A | + |
| PM36 | 2005 | Hong Kong | Blood | M/48 | + | + |
| PM37 | 2005 | Hong Kong | Blood | F/48 | − | − |
| PM38 | 2006 | Hong Kong | Blood | M/46 | + | − |
| PM39 | 2006 | Hong Kong | Blood | F/52 | − | − |
| PM40 | 2006 | Hong Kong | Blood | M/37 | + | + |
| PM41 | 2006 | Vietnam | Blood | M/30 | + | − |
| PM42 | 2006 | Vietnam | Blood | M/34 | + | − |
| PM43 | 2006 | China | Blood | M/35 | + | − |
| PM44 | 2006 | Philippines | Blood | F/44 | + | − |
| PM45 | 2007 | Indonesia | Blood | F/35 | + | − |
| PM46 | 2007 | Hong Kong | Blood | M/53 | N/A | − |
| PM47 | 2008 | Hong Kong | Blood | N/A | N/A | − |
| PY1 | N/A | Hong Kong | Blood | N/A | N/A | − |
| PY2 | N/A | Hong Kong | Blood | N/A | N/A | − |
| Q1 | N/A | Hong Kong | Blood | N/A | N/A | − |
| Q2 | N/A | Hong Kong | Blood | N/A | N/A | − |
| Q3 | N/A | Hong Kong | Blood | N/A | N/A | − |
| Q4 | N/A | Hong Kong | Blood | N/A | N/A | − |
| Q5 | N/A | Hong Kong | Blood | N/A | N/A | + |
| Q6 | N/A | Hong Kong | Blood | N/A | N/A | − |

^a^ N/A, not available; LN, lymph node; BAL, bronchoalveolar lavage; M, male; F, female; +, positive; −, negative.
