# Supplementary material for: Novel Partitivirus Enhances Virulence of and Causes Aberrant Gene Expression in Talaromyces marneffei
Source: mBio. 2018 Jun 12;9(3):e00947-18. doi: 10.1128/mBio.00947-18 (PMC6016240; doi:10.1128/mBio.00947-18)
Supplement: TABLE S3 [file mbo003183923st3.docx]

**Table S3. Transcriptional changes and predicted function of the 16 differential expressed genes in TmPV1 infected *T. marneffei* PM1**

| Gene locus tag | log_2_(FC) ^a^ | Protein annotation^b^ | Biological processes^c^ |
| --- | --- | --- | --- |
| **GABA shunt** | | | |
| GQ26_0530220 | ↑1.13209 | γ-aminobutyrate transaminase (GABA-T) | gamma-aminobutyric acid catabolism |
| **Transcription regulation** | | | |
| GQ26_0160760 | ↑2.59977 | homeobox transcription factor, putative | regulation of transcription |
| GQ26_0550320 | ↑1.54921 | C6 finger domain protein, putative | transcription |
| **Transporters** | | | |
| GQ26_0640100 | ↑1.84209 | Nitrate transporter | nitrate assimilation  nitrate transport |
| GQ26_0024190 | ↓2.65928 | Thiamine pathway transporter Thi73p | anion transport source/transmembrane transport |
| **Nitrate/nitrite metabolism** | | | |
| GQ26_0640090 | ↑1.84209 | Nitrite reductase | nitrate assimilation  nitrogen compound metabolic process |
| **Polysaccharide/cell wall metabolism** | | | |
| GQ26_0161130 | ↑1.05364 | α-galactosidase D | glycoside catabolic process  glycosylceramide catabolic process  mannan catabolic process  oligosaccharide metabolic process |
| GQ26_0121670 | ↓1.20505 | endo-1,3(4)-beta-glucanase | carbohydrate metabolism |
| **Amino acid/protein metabolism** | | | |
| GQ26_0251000 | ↓1.29855 | aspartyl protease BAR1 | adaptation of signalling pathway by response to pheromone involved in conjugation with cellular fusion protein/peptide catabolism |
| **Mp1p-like protein** | | | |
| GQ26_1170010 | ↓2.67745 | Mp1p-like protein 13 | Cell wall mannoprotein 1-like protein |
| **Hypothetical proteins** | | | |
| GQ26_0091940 | ↑1.51395 | Structural maintenance of chromosomes protein 4 | - |
| GQ26_0550330 | ↑1.54921 | Uncharacterized protein YDR124W | - |
| GQ26_1820010 | ↑1.4237 | Hypothetical protein | - |
| GQ26_1850010 | ↑1.34583 | Hypothetical protein | - |
| GQ26_0530230 | ↑1.13209 | Hypothetical protein | - |
| GQ26_0023690 | ↓2.17281 | Hypothetical protein | - |

^a^ FC, fold-change; ↑, upregulated in TmPV1 infected PM1; ↓, downregulated in TmPV1 free PM1

^b^ Annotations based on the reference genome data of PM1, BLASTX and conserved domain search results.

^c^ Biological processes based on the gene ontology terms as determined by UniProt and manual annotation.
