# Supplementary material for: Novel Partitivirus Enhances Virulence of and Causes Aberrant Gene Expression in Talaromyces marneffei
Source: mBio. 2018 Jun 12;9(3):e00947-18. doi: 10.1128/mBio.00947-18 (PMC6016240; doi:10.1128/mBio.00947-18)
Supplement: TABLE S4 [file mbo003183923st4.docx]

**Table S4. Primers used for qRT-PCR of differentially expressed and RNAi-related genes.**

| Name of Primer | Sequence (5’ – 3’) | Gene Target |
| --- | --- | --- |
| LPW33362 | AGAACAACGCTGTCTCCAGG | GABA transaminase (qRT-PCR) |
| LPW33363 | GGAGTGGTCAGGTTCCAGTG |  |
| LPW36943 | AGTGTTGGTATGGCTGTCCG | Nitrite reductase (qRT-PCR) |
| LPW36944 | AGACCGAAGCTGTATTCCGT |  |
| LPW36945 | CTGGTCACCACATCCTCTGG | Nitrate transporter (qRT-PCR) |
| LPW36946 | AAACCAGCAGCTAGGGCATT |  |
| LPW13343 | TTTACGGGACGTAAATGGCGGCCTA | *dcl-1* (qRT-PCR) (Lau *et al.*, 2013) |
| LPW13344 | AATTCTAGGCGCTGGTAAGTCGGC |  |
| LPW13347 | GTGTGAAGTGATATTGCCAAAGGG | *dcl-2* (qRT-PCR) (Lau *et al.*, 2013) |
| LPW13348 | CATTTTGTAACGGTTCAGCTGGAG |  |
| LPW14804 | GCCTCATCAAAATCCCCGGT | *qde-2* (qRT-PCR) (Lau *et al.*, 2013) |
| LPW14805 | GGAGAAACGACGACACCCAT |  |
| LPW20631 | GAACGTGAAATCGTCCGT | Actin (qRT-PCR reference) (Lau *et al.*, 2013) |
| LPW20160 | AGCAAGAATGGAACCACC |  |
